# Ligand-triggered de-repression of *Arabidopsis* heterotrimeric G proteins coupled to immune receptor kinases

**DOI:** 10.1038/s41422-018-0027-5

**Published:** 2018-03-15

**Authors:** Xiangxiu Liang, Miaomiao Ma, Zhaoyang Zhou, Jinlong Wang, Xinru Yang, Shaofei Rao, Guozhi Bi, Lin Li, Xiaojuan Zhang, Jijie Chai, She Chen, Jian-Min Zhou

**Affiliations:** 1 State Key Laboratory of Plant Genomics, Institute of Genetics and Developmental Biology, Chinese Academy of Sciences, 100101 Beijing, China; 2 State Key Laboratory of Plant Genomics, Institute of Microbiology, Chinese Academy of Sciences, 100101 Beijing, China; 3 University of Chinese Academy of Sciences, 100049 Beijing, China; 4 Center for Plant Biology, School of Life Sciences, Tsinghua University, 100084 Beijing, China; 5 National Institute of Biological Sciences, 102206 Beijing, China

## Abstract

*Arabidopsis* heterotrimeric G proteins regulate diverse processes by coupling to single-transmembrane receptors. One such receptor is the FLS2 receptor kinase, which perceives bacterial flagellin epitope flg22 to activate immunity through a class of cytoplasmic kinases called BIK1/PBLs. Unlike animal and fungal heterotrimeric G proteins that are activated by a ligand-induced guanine nucleotide exchange activity of seven-transmembrane G protein-coupled receptors (GPCRs), plant heterotrimeric G proteins are self-activating. How plant receptors regulate heterotrimeric G proteins in response to external ligands remains unknown. Here we show that RGS1, a GTPase accelerating protein, maintains *Arabidopsis* G proteins in an inactive state in complex with FLS2. Activation of FLS2 by flg22 induces a BIK1/PBL-mediated phosphorylation of RGS1 at Ser428 and Ser431 and that promotes RGS1 dissociation from the FLS2-G protein complex. This relieves G proteins from the RGS1-mediated repression and enables positive regulation of immune signaling. We additionally show that RGS1 is similarly regulated by multiple immune receptors. Our results uncover ligand-induced de-repression as a mechanism for G protein signaling in plants that is distinct from previously reported mechanism underlying the activation of heterotrimeric G proteins in other systems.

## Introduction

Heterotrimeric G proteins are universal signaling modules in eukaryotic organisms, including animals, plants and fungi. They regulate transmembrane signaling by coupling to cell surface-localized receptors. The animal and fungal heterotrimeric G proteins are directly regulated by seven-transmembrane G protein-coupled receptors (GPCRs). In the resting state, a GDP-bound Gα associates with a Gβγ dimer to form an inactive trimer. Upon stimulation by extracellular ligands, GPCRs act as guanine nucleotide exchange factors (GEFs) to promote GDP-GTP exchange in Gα to activate the G proteins. The GTP-bound Gα dissociates from the Gβγ dimer, and each entity goes on to regulate different downstream target referred to as “effectors”. Hydrolysis of GTP by the intrinsic GTPase activity of Gα allows cycling of Gα back to the GDP-bound resting state.^1,2^ The GTP hydrolysis is enhanced by the regulator of G protein signaling protein (RGS), a GTPase accelerating protein (GAP).^2,3^

The *Arabidopsis* genome encodes four Gα proteins (GPA1, XLG1, XLG2, and XLG3), one Gβ protein (AGB1), and three Gγ proteins (AGG1, AGG2, and AGG3).^4^ Among these, GPA1 is a canonical Gα, whereas XLG1, XLG2, and XLG3 are non-canonical Gα proteins that contain an N-terminal domain of unknown function in addition to the C-terminal Gα domain. Plant Gα proteins also undergo GDP- and GTP-bound cycle, and the GTPase activity of plant Gα proteins is similarly enhanced by RGS proteins. *Arabidopsis* contains a single RGS1 protein that negatively regulates GPA1-mediated signaling through its GAP activity.^5,6^ Plant heterotrimeric G proteins have been shown to associate with receptor kinases (RKs), receptor-like kinases (RLKs), or receptor-like proteins (RLPs), which are all single-transmembrane proteins. The maize RLP FEA2 associates with the Gα protein CT2 to maintain shoot apical meristem.^7^ The soybean RK NFR1 interacts with Gα proteins to control nodulation.^8^ The *Arabidopsis* RK ERECTA genetically interacts with heterotrimeric G protein components to regulate disease resistance.^9,10^ We have shown recently that plant heterotrimeric G proteins are associated with and regulated by immune receptor kinase FLS2.^11^ However, these plant receptors are not known to act as GEFs. Moreover, plant Gα proteins are self-activating and can bind GTP in the absence of GEFs.^12,13^ How plant receptor kinases regulate heterotrimeric G proteins remains poorly understood.

Immune RKs FLS2, EFR, LYK5, and PEPRs are pattern recognition receptors (PRRs) that recognize microbe- or plant-derived molecular patterns including the bacterial flagellin epitope flg22, elongation factor Tu epitope elf18, fungal cell wall component chitin, and plant elicitor peptides (Peps), triggering a series of immune responses culminated in disease resistance against diverse pathogens.^14–17^ Among these, FLS2 has been extensively studied and serves as a model to understand RK-mediated signaling, particularly the regulation of heterotrimeric G proteins. Upon flg22 binding, FLS2 rapidly recruits its co-receptor BAK1, a receptor-like kinase, to form an active receptor complex and initiates immune signaling.^18,19^ Downstream of FLS2, receptor-like cytoplasmic kinase family VII (RLCKVII) members BIK1 and PBS1-Like (PBL) kinases regulate various downstream components to activate defense responses.^20–23^

Heterotrimeric G proteins play an important role in RK-mediated immune signaling. XLG2/3, AGB1, and AGG1/2 are required for mesophyll immunity mediated by RKs, including FLS2.^24–26^ We recently showed that the XLG2-Gβγ complex interacts with the FLS2-BIK1 receptor complex and positively regulates immunity.^11^ In the resting state, XLG2-Gβγ complex directly interacts with and promotes the stability of BIK1. XLG2 additionally regulates immune signaling independent of BIK1 stability, as restoration of BIK1 accumulation in the *xlg2* mutant only partially restores FLS2-mediated immune signaling and disease resistance. Furthermore, GPA1 is genetically required for FLS2-mediated stomatal defense,^27,28^ although the underlying mechanism remains unknown.

Several receptor-like kinases have been shown to phosphorylate RGS1, and this has been reported to promote RGS1–GPA1 dissociation and RGS endocytosis,^29,30^ or enhance the GAP activity.^8^ We recently showed that upon flg22 treatment, XLG2 dissociates from AGB1 and the FLS2 receptor complex, suggesting a ligand-induced activation of XLG2-Gβγ. However, it is not clear whether the observed XLG2–AGB1 dissociation reflects a change in XLG2 guanine nucleotide-binding states. If the answer is yes, it is not known how a self-activating plant Gα protein is maintained in an inactive state prior to ligand perception and how the ligand perception regulates the guanine nucleotide-binding states of XLG2 and activation of the XLG2-Gβγ heterotrimer.

Here we show that flg22 activates XLG2-Gβγ by regulating guanine nucleotide-binding states. We further show that RGS1 regulates heterotrimeric G proteins in the FLS2 receptor complex through a direct interaction with FLS2 and XLG2. Prior to flg22 perception, RGS1 maintains XLG2-Gβγ in an inactive state in the receptor complex through its GAP activity. Upon activation by flg22, RGS1 is phosphorylated by BIK1/PBLs, and the phosphorylation triggers its dissociation from both FLS2 and XLG2. This enables auto-activation of Gα protein, which then dissociates from the Gβγ dimer and FLS2 receptor complex to promote immune signaling. We further provide evidence that elf18, chitin and Pep2 similarly regulate RGS1 phosphorylation and that flg22 similarly regulates GPA1 activation through RGS1. Our findings illustrate how a plant RK activates heterotrimeric G proteins through a RGS1-dependent de-repression and provide a paradigm for plant RK-mediated regulation of heterotrimeric G proteins.

## Results

### Guanine nucleotide-binding is required for Gα–Gβ interactions and plant immune signaling

**Fig. 1:**
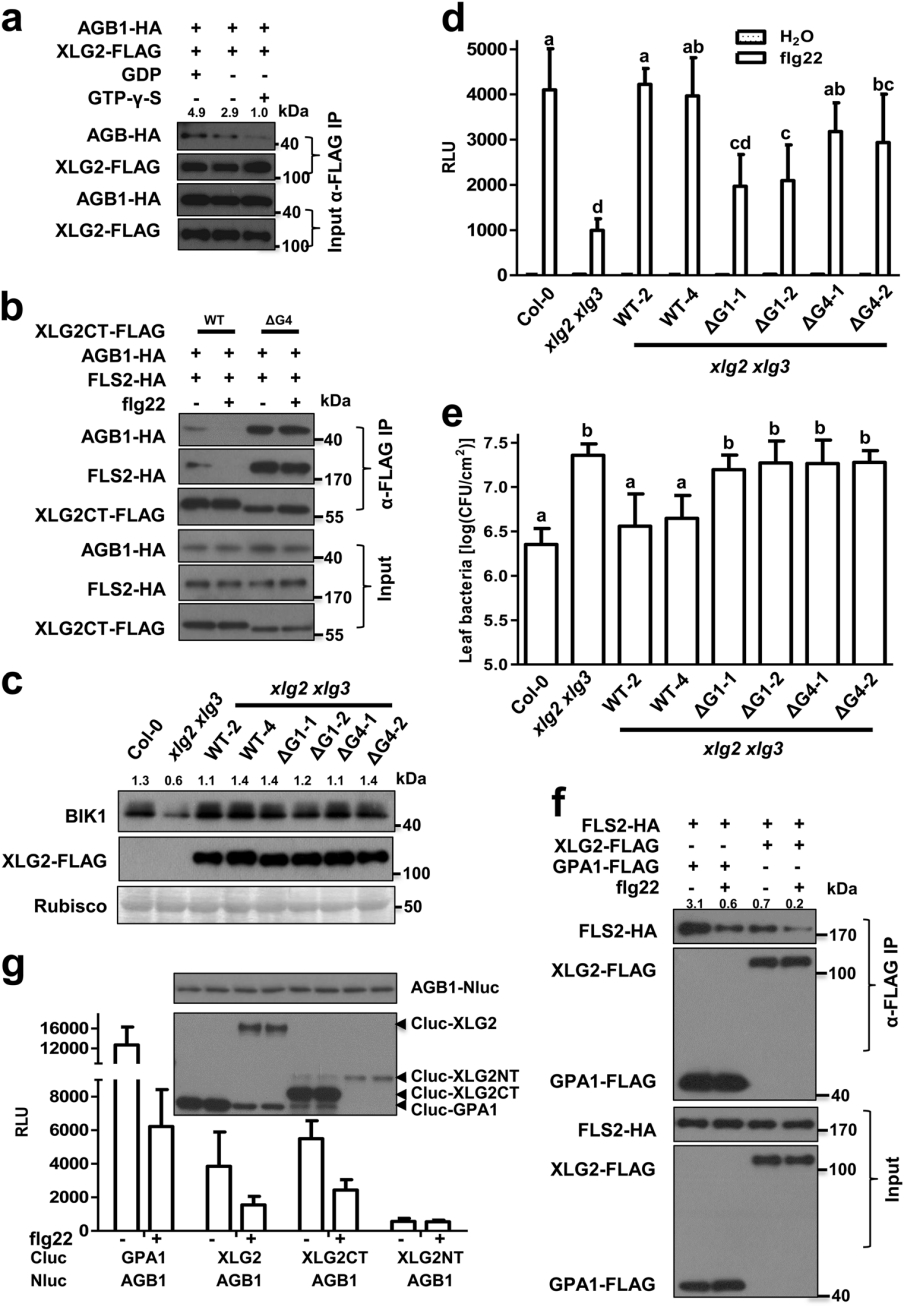
Guanine nucleotide-binding regulates XLG2–AGB1 interaction and FLS2-mediated immune signaling. **a** GDP enhances, whereas GTP-γ-S impairs XLG2–AGB1 interaction. The indicated constructs were transfected into Col-0 protoplasts, and co-IP assays were performed in the presence of the indicated nucleotides. Note that endogenous AGGs are likely essential for the interaction. Numbers indicate arbitrary densitometry units normalized to input AGB1-HA. **b** XLG2 mutant defective in guanine nucleotide-binding fails to dissociate from AGB1 in response to flg22 treatment. The indicated constructs were transfected into Col-0 protoplasts, treated with or without flg22, and co-IP was performed. **c** Nucleotide-binding motifs are not required for XLG2-mediated control of BIK1 stability. Protein samples were isolated from experiment described in Fig. 1d and subjected to anti-BIK1 immunoblot analyses. **d** Nucleotide-binding motifs of XLG2 are required for immune signaling. *xlg2 xlg3* plants complemented with WT *XLG2* (WT), *XLG2ΔG1* (ΔG1), or *XLG2ΔG4* (ΔG4) transgenes were tested for flg22-induced H_2_O_2_ production. Different letters indicate significant difference at *P* < 0.05 (mean ± SD, *n* ≥ 6, one-way ANOVA followed by Tukey’s post hoc test). **e** Nucleotide-binding motifs of XLG2 are required for resistance to *Pst* DC3000. The indicated plants were infiltrated with *Pst* DC3000, and bacterium number was determined 3 days later. Different letters indicate significant difference at *P* < 0.05 (mean ± SD, *n* ≥ 6, one-way ANOVA followed by Tukey’s post hoc test). **f** Flg22 treatment destabilizes GPA1–FLS2 and XLG2–FLS2 interactions. Col-0 protoplasts expressing the indicated constructs were treated with flg22, and total protein was subjected to co-IP assays. **g** Flg22 treatment destabilizes GPA1–AGB1 and XLG2–AGB1 interactions. The indicated constructs were expressed in *N. benthamiana*, and luciferase complementation assays were performed. Relative luminescence units (RLU) indicate levels of protein–protein interactions. (RLU, means ± SD; *n* ≥ 6). Numbers on top of the gel blots indicate arbitrary densitometry units of co-IP products normalized to the amounts of Rubisco protein (**c**) and FLS2-HA (**f**). The experiments were performed two (**a**, **c**) or three (**b**, **d**–**g**) times with similar results

While the flg22-induced XLG2–AGB1 dissociation suggests a ligand-induced activation of XLG2-Gβγ, this hypothesis has not been rigorously tested. We first sought to test whether the XLG2–AGB1 interaction is stabilized in the presence of GDP and diminished in the presence of GTP-γ-S, a non-hydrolyzable form of GTP, in co-IP assays. While addition of GDP increased XLG2–AGB1 interaction by ~70% compared to control, addition of GTP-γ-S diminished the interaction to 34% of the control (Fig. 1a). These data are consistent with the expected role of GDP- and GTP-bound XLG2 in XLG2-Gβγ activation.

GPA1 contains five guanine-nucleotide binding motifs, G1, G2, G3, G4, and G5. XLG2 also contains putative guanine-nucleotide binding motifs in the C-terminal Gα domain. Among these, G1, G2, and G4, but not G3 and G5, are conserved with their counterparts in canonical Gα proteins (Supplementary information, Figure S1a).^12^ We reasoned that if XLG2-Gβγ is activated through GTP-binding, disruption of the guanine nucleotide-binding motifs should stabilize the XLG2–AGB1 interaction and inhibit the XLG2-mediated immune signaling. We individually deleted G1–G4 motifs in either full-length or C-terminal Gα domain (XLG2CT) of XLG2 and examined their interactions with AGB1. These deletions markedly enhanced the interactions of both the full-length XLG2 and XLG2CT with AGB1 (Fig. 1b; Supplementary information, Figure S1b and c). Importantly, while the XLG2CT–AGB1 interaction was diminished following flg22 treatment, the XLG2CTΔG4–AGB1 interaction was not affected (Fig. 1b). Similarly, the XLG2ΔG1 and XLG2ΔG4 mutants displayed stronger interactions with FLS2 (Supplementary information, Figure S1d). These results indicate that the guanine nucleotide-binding motifs are indeed required for the FLS2-mediated regulation of XLG2–AGB1 interaction, supporting that the guanine nucleotide-binding state of XLG2 is important for the stability of XLG2-Gβγ heterotrimer in the FLS2 receptor complex upon flg22 treatment.

We further tested a role of the guanine nucleotide-binding motifs in plant immunity by complementing the *xlg2 xlg3* mutant with FLAG-tagged wild-type (WT) *XLG2, XLG2ΔG1*, and *XLG2ΔG4* transgenes under the control of *XLG2* native promoter. An examination of BIK1 protein levels in these transgenic lines showed that the WT and the two mutant forms of *XLG2* transgenes all restored BIK1 accumulation to the Col-0 level, indicating that guanine nucleotide-binding is not required for XLG2-mediated control of BIK1 stability (Fig. 1c). As we previously reported, the *xlg2 xlg3* mutant was severely impaired in flg22-induced H_2_O_2_ production (Fig. 1d). The WT *XLG2* transgene fully restored flg22-induced H_2_O_2_ production, whereas the two *XLG2ΔG* mutant transgenes only partially restored H_2_O_2_ production (Fig. 1d), indicating that guanine nucleotide-binding is partially required for XLG2-mediated ROS production. We further inoculated these transgenic plants with a virulent bacterial strain *Pseudmonas syringae* pv *tomato* DC3000 (*Pst*) and examined bacterial growth. The *xlg2 xlg3* plants were highly susceptible to the bacteria and supported ~10-fold more bacterial growth (Fig. 1e). The transgenic lines carrying the WT *XLG2* transgene were largely similar to Col-0 plants, whereas the transgenic lines carrying the *XLG2ΔG* mutants were most similar to the non-transgenic *xlg2 xlg3* mutant plants, indicating that guanine nucleotide-binding is required for XLG2-mediated resistance to virulent bacteria. Although the *xlg2 xlg3* mutant is severely compromised in disease resistance to *Pst* bacteria, flg22-induced ROS production, and BIK1 stability,^11^ the *bik1* mutant is not compromised in disease resistance to virulent *Pst* bacteria and is only partially impaired in flg22-induced ROS production.^21,31^ Thus the reduced BIK1 stability does not appear to explain the compromised disease resistance to *Pst* bacteria and only partially explains the impaired ROS production in the *xlg2 xlg3* mutant.^11,21^ The near *xlg2 xlg3* level susceptibility to *Pst* bacteria and partial restoration of ROS production by the *XLG2ΔG* mutants were consistent with our proposal that XLG2 additionally regulates immunity independent of BIK1 stability.^11^ Together, these results supported that, in addition to stabilizing BIK1 in the resting state, XLG2 contributes to immunity after ligand-induced activation in a manner dependent on guanine nucleotide binding.

Although the canonical Gα protein GPA1 is dispensable for flg22-induced ROS production and disease resistance in mesophyll tissue,^24,26,27^ it plays a role in FLS2-mediated stomatal defense.^27,28^ We asked whether GPA1 is also coupled to FLS2. Co-IP assays showed that GPA1 indeed interacted with FLS2, and the interaction was diminished upon flg22 treatment (Fig. 1f). Luciferase complementation assays in *Nicotiana benthamiana* plants further showed that the GPA1–AGB1, XLG2–AGB1, and XLG2CT–AGB1 interactions were similarly destabilized upon flg22 treatment (Fig. 1g), indicating that GPA1-Gβγ is also activated upon flg22 perception, which is consistent with a previous study.^29^ The results additionally showed that the ligand-triggered XLG2-Gβγ activation does not require the N-terminal domain. We further tested a role of guanine nucleotide-binding motif in GPA1–AGB1 interaction. GPA1ΔG1 and GPA1ΔG4 showed much stronger interaction with AGB1 than did the WT GPA1 (Supplementary information, Figure S1e), indicating that the guanine nucleotide-binding motifs are required for the regulation of both XLG2–AGB1 and GPA1–AGB1 interactions. We conclude that guanine nucleotide-binding motifs negatively regulate the stability of XLG2-Gβγ/GPA1-Gβγ heterotrimers.

### RGS1 negatively regulates XLG2- and GPA1-mediated immune signaling

**Fig. 2:**
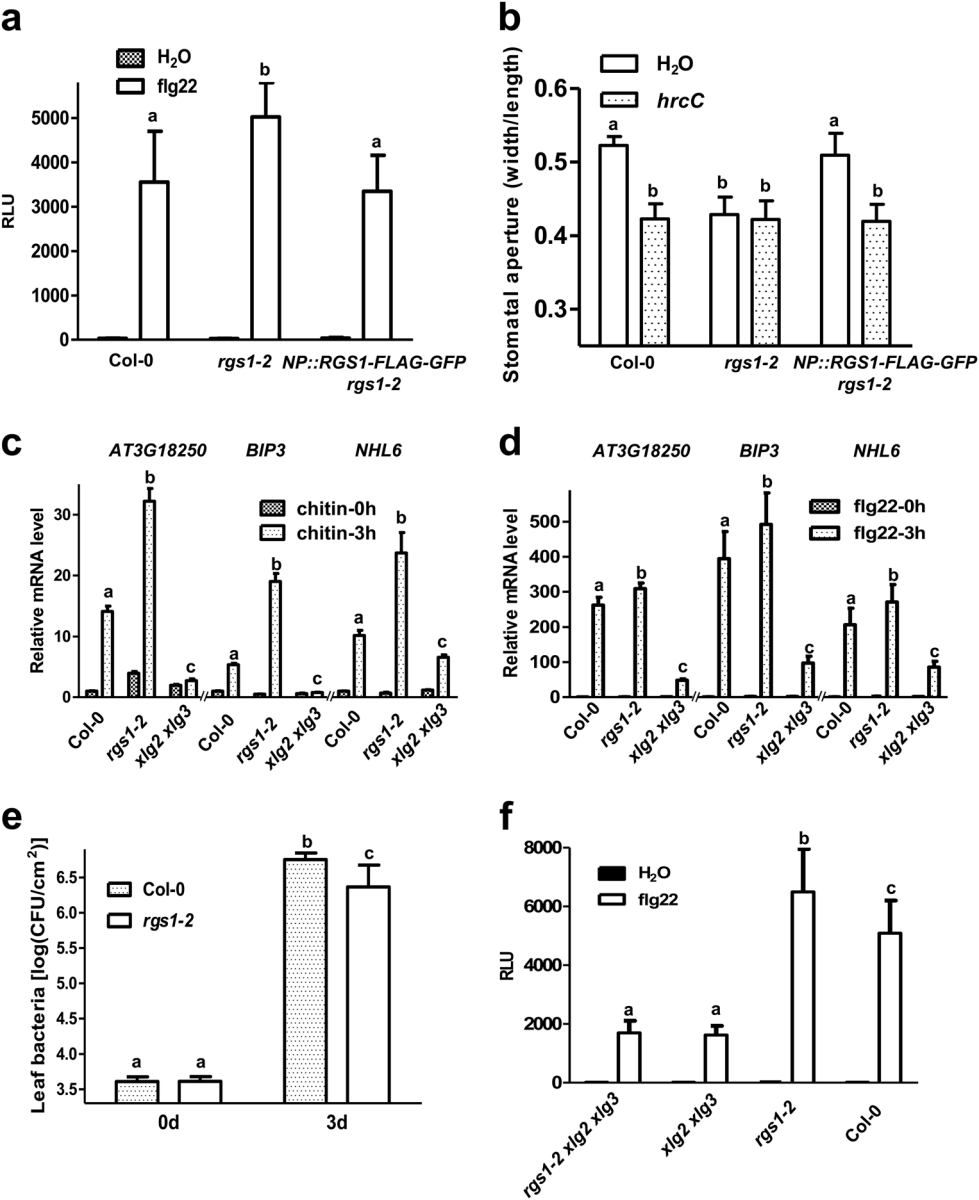
RGS1 negatively regulates XLG2-mediated immune signaling and disease resistance to a bacterial pathogen. **a** *rgs1-2* displays enhanced ROS production in response to flg22. The *rgs1-2* line was stably transformed with *RGS1-FLAG-GFP* under the control of *RGS1* native promoter. A T2 transgenic line and the *rgs1-2* mutant were tested for flg22-induced H_2_O_2_ production. Different letters indicate significant difference at *P* < 0.05 (mean ± SD, *n* ≥ 6, one-way ANOVA followed by Tukey’s post hoc test). **b** *rgs1-2* displays reduced stomatal aperture and is insensitive to *Pst* DC3000 *hrcC*^*−*^ treatment. The indicated genotypes were subjected to stomata measurement assay and the stomata opening is showed by calculating the ratio of width to length. (mean ± SD, *n* ≥ 6, one-way ANOVA followed by Tukey’s post hoc test). **c**, **d** *rgs1-2* mutant shows enhanced defense gene expression upon flg22 (**c**) and chitin (**d**) treatment. Leaves of Col-0, *xlg2 xlg3*, and *rgs1-2* plants were infiltrated with flg22 or chitin for 3 h, and total RNA were extracted and subjected to qPCR analysis of the indicated genes. Different letters indicate significant difference at *P* < 0.05 (mean ± SD, *n* ≥ 6, one-way ANOVA followed by Tukey’s post hoc test). **e** *rgs1-2* displays increased resistance to *Pseudomonas syringae* pv *tomato* (*Pst*) bacteria. Plants of the indicated genotypes were inoculated by spray with *Pst* DC3000, and bacterial population in the leaf was determined at 0 and 3 days after inoculation. Different letters indicate significant difference at *P* < 0.01 (mean ± SD, *n* ≥ 6, one-way ANOVA followed by Tukey’s post hoc test). **f** XLG2/XLG3 act downstream of RGS1 to regulate immune signaling. Plants of the indicated genotypes were examined for flg22-induced H_2_O_2_ production. Different letters indicate significant difference at *P* < 0.05 (mean ± SD, *n* ≥ 6, one-way ANOVA followed by Tukey’s post hoc test). The experiments were performed two (**a**, **c**, **d**) or three (**b**, **e**, **f**) times with similar results

We next sought to determine how flg22 regulates G protein activation. RGS1 is known to negatively regulate GPA1-mediated control of cell proliferation and sugar signaling.^5,6^ Two recent reports suggested that RGS1 positively regulate flg22-induced H_2_O_2_ production,^32,33^ which contradicts the predicted role of RGS1 in regulating GPA1 and XLG2. We identified two *rgs1* mutant alleles, *rgs1-1* and *rgs1-2*, both abolished in the accumulation of intact *RGS1* transcripts (Supplementary information, Figure S2a). Consistent with previous report, the *rgs1-1* and *rgs1-2* mutant plants are slightly bigger in size than the Col-0 plants but are otherwise normal when grown under short-day conditions. (Supplementary information, Figure S2b).^5,34^ Contrary to previous reports, both alleles reproducibly displayed significantly increased H_2_O_2_ production in response to flg22 treatment (Fig. 2a; Supplementary information, Figures S2c). The *rgs1* alleles similarly displayed increased H_2_O_2_ production in response to chitin (Supplementary information, Figure S2d). Because our *rgs1-2* line was independently identified from a segregating population, the mutant lines in different laboratories may carry different mutations unrelated to *RGS1*. We therefore complemented *rgs1-2* plants with the *RGS1-FLAG-GFP* transgene under the control of *RGS1* native promoter. The resulting transgenic plants showed a Col-0 level ROS production in response to flg22 treatment (Fig. 2a), indicating that RGS1 indeed negatively regulates flg22 signaling.

We further examined stomatal aperture in these plants following treatment of *Pst hrcC*^*−*^ mutant strain, which lacks a functional type III secretion system needed to secrete immune-suppressing virulence proteins and thus provides a means to measure pattern-triggered immunity.^35^ Strikingly, while the Col-0 stomata were fully open and responded to *Pst hrcC*^*−*^ bacteria treatment by reducing stomatal aperture, the *rgs1-2* mutant displayed greatly reduced stomatal aperture before treatment and failed to respond to the bacterial treatment (Fig. 2b; Supplementary information, Figure S2e). The *rgs1-2* line complemented with the *RGS1-FLAG-GFP* transgene was nearly identical to Col-0 plants in stomatal aperture and responsiveness to the bacterial treatment. Nevertheless, the *gpa1-3* mutant showed greater stomatal aperture and is insensitive to *Pst hrcC*^*−*^ bacterium-induced stomata closure (Supplementary information, Figure S2e).^27,28^ In contrast, the *xlg2 xlg3* mutant showed normal stomatal aperture and responsiveness to *Pst hrcC*^*−*^ bacteria, indicating that XLG2 is not required for stomatal defense. Thus the defects of *rgs1* in stomatal aperture are consistent with a constitutive activation of GPA1, but not XLG2.

To further substantiate our findings, we sought to determine other immune responses triggered by microbial patterns. The flg22 and chitin-induced activation of MAP kinases (MPKs) was normal in both *rgs1-2* and *xlg2 xlg3* mutants (Supplementary information, Figure S2f), suggesting that the G protein signaling is not involved in flg22- and chitin-induced MPK activation. In a separate study we performed RNA-seq analysis in order to identify flg22-induced transcripts that are affected in the *xlg2 xlg3* mutant (work in progress). Three chitin- and/or flg22-induced genes, including *BIP3, NHL6*, and *At3G18250*, that showed the greatest defects in *xlg2 xlg3* plants were selected and confirmed by quantitative RT-PCR analyses. The expression of all these three genes were significantly down-regulated in the *xlg2 xlg3* mutant as compared to that in Col-0 plants (Fig. 2c, d). In contrast, their expression in response to chitin and/or flg22 was significantly increased in the *rgs1-2* mutant. We further examined *Pst* bacterial growth in plants and found that the *rgs1-2* supported significantly less bacterial growth compared to Col-0 plants (Fig. 2e), indicating that RGS1 negatively regulates immunity to *Pst* bacteria.

The aforementioned results are all consistent with the expected negative role of RGS1 in G protein signaling.^5,6,8,34,36,37^ To test genetic relationship between *RGS1* and *XLG2/3*, we constructed an *rgs1 xlg2 xlg3* triple mutant. The triple mutant and *xlg2 xlg3* double mutant showed indistinguishable flg22-triggered ROS production that was only ~30% of the Col-0 level (Fig. 2f). Together these results demonstrate that RGS1 negatively regulates immune signaling upstream of XLG2/3.

### RGS1 dynamically interacts with XLG2 and multiple PRRs

**Fig. 3:**
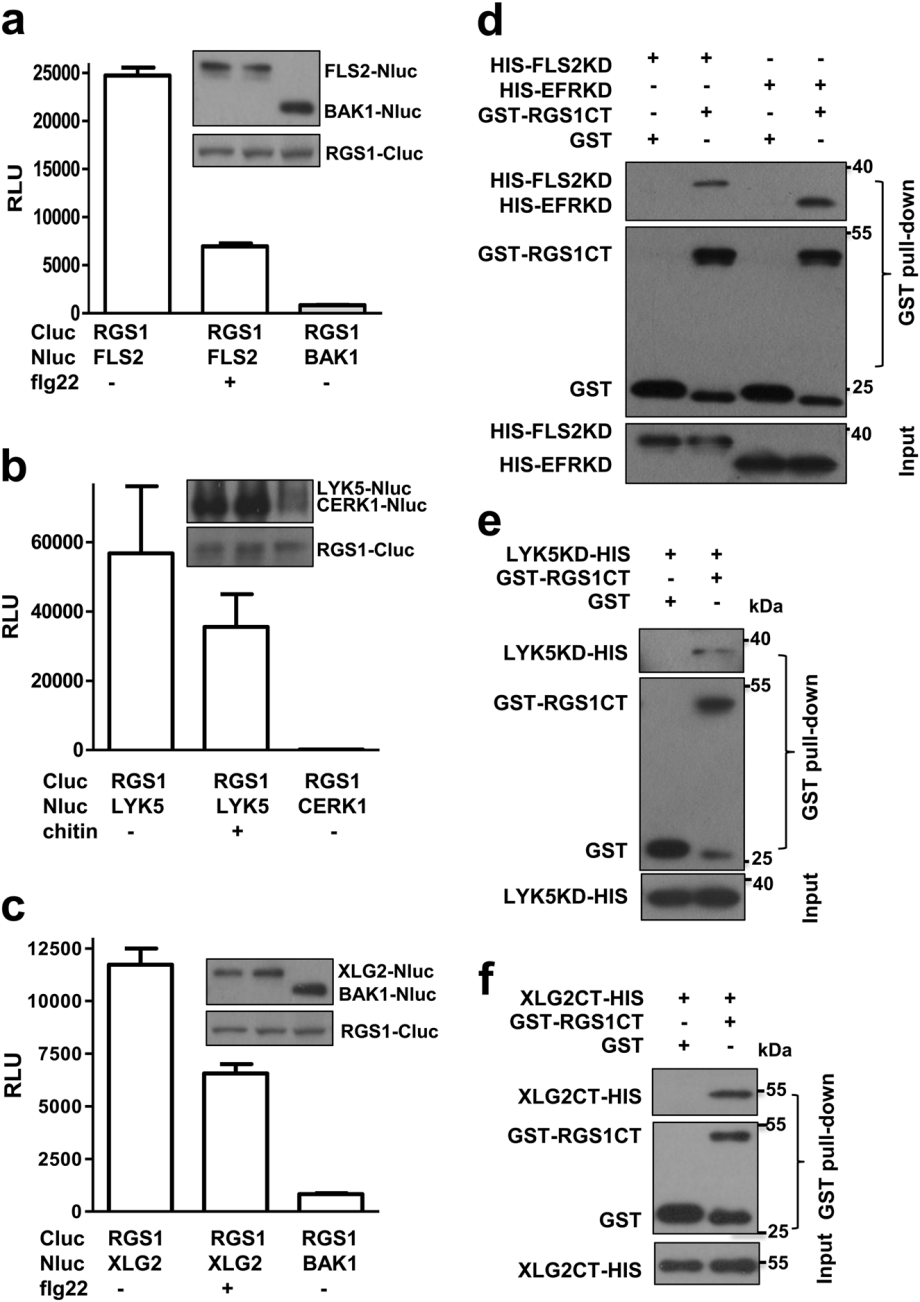
RGS1 dynamically interacts with XLG2 and multiple PRRs. (**a-c**) RGS1 dynamically interacts with FLS2 (**a**), LYK5 (**b**), and XLG2 (**c**). Luciferase complementation assays were performed on *Nicotiana benthamiana* plants by *Agrobacterium*-mediated transient expression of the indicated constructs. Relative strength of protein–protein interactions is expressed as arbitrary relative luminescence units (RLU) (mean ± SD; *n* ≥ 6). Immunoblots show levels of protein accumulation. **d** RGS1 C-terminus (RGS1CT) interacts with FLS2 kinase domain (FLS2KD) and EFR kinase domain (EFRKD) in vitro. GST pull-down assays were performed with the indicated recombinant proteins. **e**,** f** RGS1 C-terminus (RGS1CT) interacts with LYK5 kinase domain (LYK5KD) (**e**) and XLG2 C-terminus (XLG2CT) (**f**) in vitro. Protein interactions were examined by GST pull-down assays. The experiments were performed three times with similar results

RGS1 contains an N-terminal seven-transmembrane domain, an RGS box and a C-terminal regulatory tail.^5^ Previous reports with various assays have suggested an interaction of RGS1 with components of receptor kinase complexes including PEPR1, BAK1, and NFR1.^8,29,30^ We performed luciferase complementation assays in *N. benthamiana* plants and co-IP assays in *Arabidopsis* protoplasts to determine whether RGS1 is associated with the FLS2, EFR, LYK5 and XLG2. Luciferase complementation assays showed that RGS1 strongly interacted with FLS2, EFR, LYK5, and XLG2, but not the co-receptors BAK1 and CERK1 (Fig. 3a–c; Supplementary information, Figure S3c). The interactions were notably weakened upon treatment with flg22, elf18, or chitin, indicating a ligand-induced dissociation. Immunoblot analyses demonstrated that the differential interaction was not caused by differential amounts of protein accumulation. Co-IP assays showed that the RGS1 C-terminus (RGS1CT), which contains an RGS box and the regulatory tail, interacted with FLS2 and XLG2 (Supplementary information, Figures S3a and b). Flg22 treatment strongly diminished the RGS1CT–FLS2 interaction and reduced the RGS1CT–XLG2 interaction. As reported previously, the full-length RGS1 was below detection limit when expressed in plants,^38^ we were unable to detect the full-length RGS1-FLAG protein in protoplasts, precluding a co-IP assay on the full-length RGS1. GST pull-down assays showed that RGS1CT interacted with FLS2 kinase domain (FLS2KD), EFR kinase domain (EFRKD), and LYK5 kinase domain (LYK5KD) (Fig. 3d, e). RGS1CT also interacted with XLG2CT in vitro (Fig. 3f). Together, these results demonstrated that RGS1 directly interacted through its C-terminus with both PRR kinase domain and XLG2 C terminal domain, and flg22 and chitin treatments destabilized these interactions.

### RGS1 GAP activity stabilizes G protein heterotrimers in the FLS2 receptor complex in the resting state

We next sought to determine mechanism by which RGS1 regulates FLS2-mediated signaling. We first asked whether RGS1 plays a role in the stability or activation of the FLS2 receptor complex. Immunoblot analyses showed that the *rgs1-2* mutant accumulated normal amounts of FLS2 and its co-receptor BAK1 compared to Col-0 plants (Supplementary information, Figure S4a). We introduced a *BIK1-HA* transgene^21^ into *rgs1-2* by crossing to facilitate detection of BIK1 in plants. Immunoblot analyses showed that BIK1 accumulation was not affected by the *rgs1* mutation (Supplementary information, Figure S4a). Co-IP assays and immunoblot analyses were performed to further test whether the *rgs1-2* mutation affects flg22-induced FLS2-BAK1 association and BIK1 phosphorylation. Col-0 and *rgs1-2* mutant protoplasts showed indistinguishable FLS2–BAK1 interaction (Supplementary information, Figure S4b) and BIK1 phosphorylation (Supplementary information, Figure S4c) after flg22 treatment. These results indicate that, RGS1 is not required for the stability nor flg22-triggered activation of the FLS2 receptor complex.

**Fig. 4:**
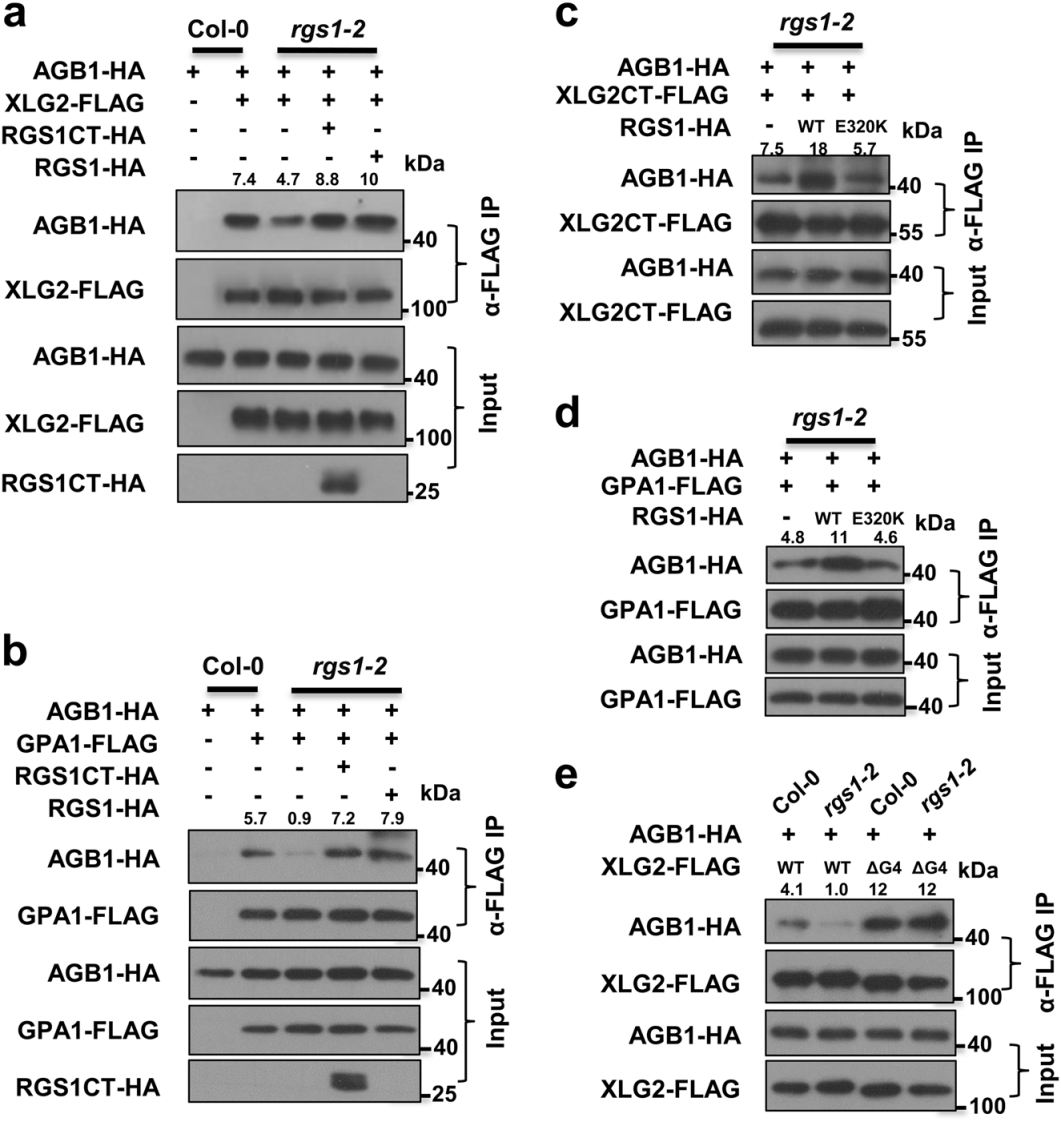
RGS1 stabilizes XLG2/GPA1–AGB1 interactions through GAP activity **a**, **b** *RGS1* is required for the stability of XLG2–AGB1 (**a**) and GPA1–AGB1 (**b**) interactions. The indicated constructs were transiently expressed in Col-0 or *rgs1-2* protoplasts, and co-IP was performed. **c**,** d** The GAP activity of RGS1 is required for the stability of XLG2–AGB1 (**c**) and GPA1–AGB1 (**d**) interactions. Co-IP assays were performed as in **a**. **e** Disruption of nucleotide-binding motifs in XLG2 stabilizes its interaction with AGB1 independent of RGS1. The indicated constructs were transiently expressed in Col-0 or *rgs1-2* protoplasts, and co-IP was performed. Numbers on top of the gel blots indicate arbitrary densitometry units of co-IP products normalized to the amounts of AGB1-HA protein. The experiments were performed twice with similar results

We next tested whether RGS1 plays a role in maintaining the stability of G protein heterotrimers in the FLS2 receptor complex in the resting state. Co-IP assay showed that the XLG2–AGB1 interaction was reduced by ~40% in *rgs1-2* compared to Col-0 (Fig. 4a). Transfection of *rgs1-2* protoplasts with RGS1CT restored the XLG2–AGB1 interaction to a level slightly higher than that in Col-0 protoplasts. Transfection with the full-length RGS1 also restored XLG2–AGB1 interaction, although the amount of RGS1 was below detection limit. The XLG2CT–AGB1 interaction was similarly impaired in *rgs1-2* protoplasts (Supplementary information, Figure S5a). Likewise, the GPA1–AGB1 interaction was nearly abolished in *rgs1-2* protoplasts, whereas transfection with RGS1 restored the interaction (Fig. 4b). We previously showed that the flg22-induced XLG2–AGB1 dissociation is accompanied by XLG2–FLS2 dissociation.^11^ Co-IP assays showed an impaired XLG2–FLS2 interaction in *rgs1-2* in the resting state (Supplementary information, Figure S5b), which is consistent with a constitutive activation of the G proteins. These results indicate that RGS1 regulates the stability of Gαβγ heterotrimers and their interaction with FLS2.

We next examined whether the GAP activity of RGS1 is required for its regulation of the G proteins by complementing *rgs1-2* mutant protoplasts with a GAP-dead form of RGS1^E320K^. Co-IP assays showed that while complementation with the WT RGS1 strongly enhanced XLG2CT–AGB1 and GPA1–AGB1 interactions, RGS1^E320K^ were completely unable to do so (Fig. 4c, d), supporting that RGS1 regulates Gαβγ stability through its GAP activity.

To further test the possibility that RGS1 stabilizes XLG2-Gβγ in the FLS2 complex through GAP activity, we tested whether disruption of guanine nucleotide-binding renders XLG2–AGB1 interaction insensitive to the *rgs1-2* mutation. Co-IP assays showed that, unlike the WT XLG2, XLG2ΔG4 interacted strongly with AGB1, and the interaction was not affected by the *rgs1-2* mutation (Fig. 4e), indicating that the guanine nucleotide-binding motif is essential for the RGS1-mediated regulation of XLG2-Gβγ heterotrimer. Together, these results support that RGS1 indeed actively arrests G proteins in the FLS2 receptor complex through its GAP activity, whereas a lack of RGS1 leads to de-repression of the G proteins.

### Patterns induce RGS1 phosphorylation at Ser431

**Fig. 5:**
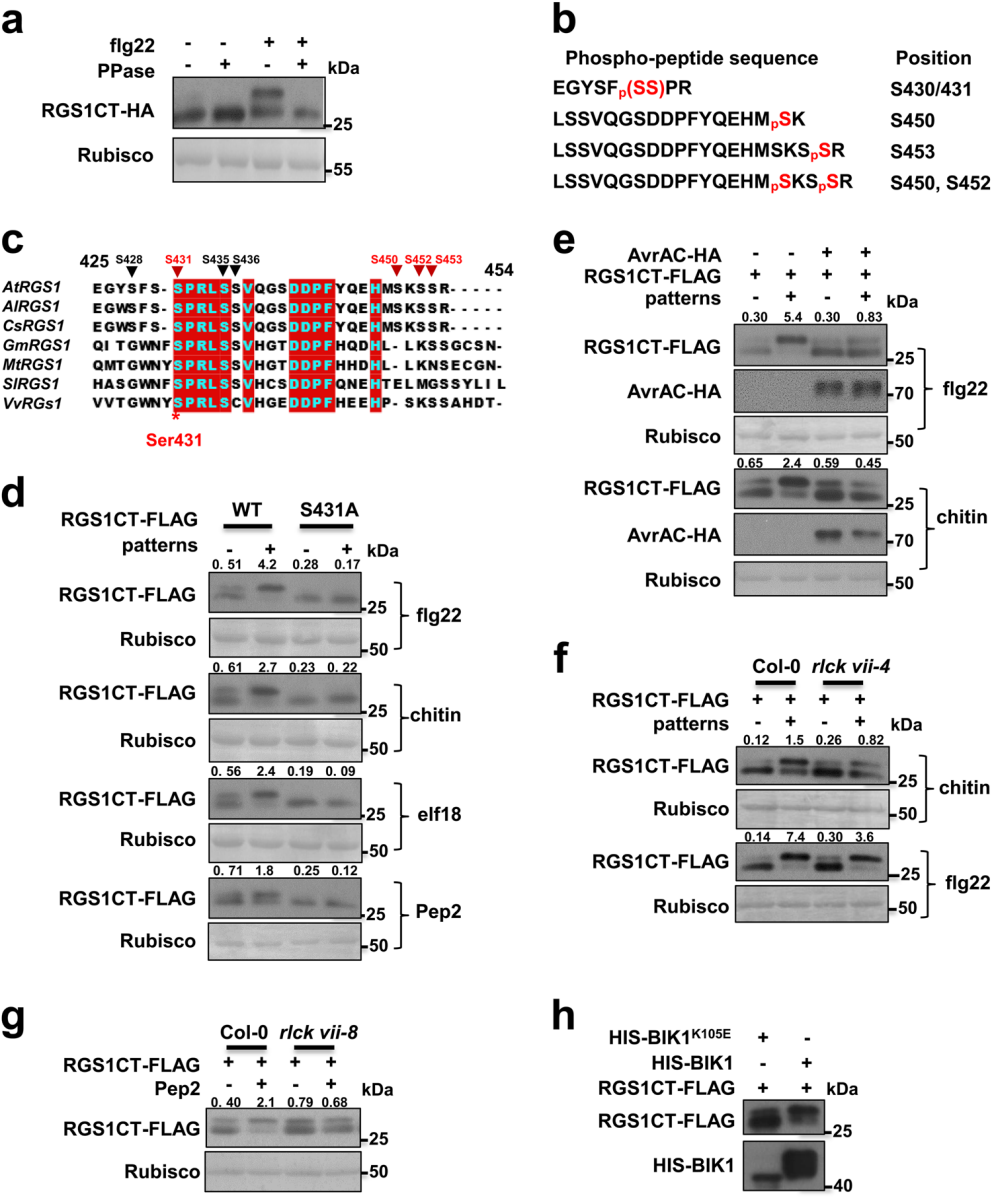
Flg22 induces RGS1 phosphorylation at Ser431. **a** Flg22 induces phosphorylation of RGS1 C-terminus (RGS1CT). Col-0 protoplasts expressing RGS1CT-HA were treated with 1 μM flg22 for 5 min before protein extraction. Protein samples were treated with (+) or without (−) λ protein phosphatase (PPase) before immunoblot analysis. **b** Phospho-peptides identified from mass spectrometric analyses. RGS1CT-FLAG was transiently expressed in protoplasts, affinity purified following flg22 treatment and subjected to LC-MS/MS analysis. **c** Alignment of the regulatory tail of RGS proteins from different plant species. At *Arabidopsis thaliana*, Al *Arabidopsis lyrata*, Cs* Camelina sativa*, Gm* Glycine max*, Mt *Medicago truncatula*, Sl *Solanum lycopersicum*, Vv *Vitis vinifera*. Position of Ser431 is indicated by an asterisk. **d** RGS1 Ser431 is phosphorylated upon flg22, elf18, chitin, and Pep2 treatment and substitution of Ser431 with Ala in RGS1CT abolishes pattern-induced phosphorylation. The indicated constructs were transiently expressed in WT protoplasts, which were then treated with indicated flg22, elf18, chitin, or Pep2, and RGS1CT protein mobility was determined by immunoblot analyses. **e** AvrAC blocks flg22- and chitin-induced RGS1 phosphorylation at Ser431. RGS1CT-FLAG were transiently expressed in WT protoplasts along with AvrAC-HA, and RGS1CT phosphorylation was detected by immunoblot. **f** Chitin-induced RGS1CT phosphorylation is compromised in *rlck vii-4* mutant. RGS1CT-FLAG was expressed in protoplasts prepared from WT and *rlck vii-4* mutant plants. After flg22 or chitin treatment of protoplasts, the protein mobility was detected by immunoblot. **g** Pep2-induced RGS1CT phosphorylation is compromised in *rlck vii-8* mutants. RGS1CT-FLAG protein mobility was examined in WT and *rlck vii-8* protoplasts. **h** BIK1 phosphorylates RGS1CT-FLAG in vitro. RGS1CT-FLAG was expressed and purified from protoplasts, incubated with HIS-tagged BIK1 and BIK1^K105E^ recombinant proteins, and RGS1CT-FLAG protein mobility was detected with immunoblot analyses. Numbers on top of the gel blots indicate ratio of phosphorylated (upper band) to non-phosphorylated (lower band) RGS1CT-FLAG. The experiments were performed two (**f**) or three (**a**,** d**,** e**,** g**, **h**) times with similar results

The aforementioned results prompted us to test the possibility that the dissociation of RGS1 from the FLS2-G protein complex is a trigger for G protein activation upon flg22 perception. During the analyses of the RGS1 protein, we consistently observed an flg22-induced band-shift of the C-terminal fragment of RGS1 (Fig. 5a). The band-shift was sensitive to phosphatase treatment, indicating that RGS1CT underwent an flg22-induced phosphorylation.

To identify phospho-sites, we transiently expressed RGS1CT-FLAG in Col-0 protoplasts and treated the protoplasts with flg22. The RGS1CT-FLAG protein was subsequently affinity-purified and subjected to LC-MS/MS analysis. The identified peptides covered 90.6% of the RGS1CT sequence (Supplementary information, Figure S6a), including four peptides containing four or five phosphorylated serine residues. Ser430 and/or Ser431 (the two residues could not be differentiated by the LC-MS/MS data), Ser450, Ser452, and Ser453 (Fig. 5b). These phospho-sites are all clustered in ~30 amino acids of the regulatory tail. Sequence alignment showed that Ser431 is conserved in all dicot species examined, whereas other residues are not (Fig. 5c). Site-directed mutagenesis showed that Ser431 is essential for the flg22-induced phosphorylation, as RGS1CT^S431A^-FLAG failed to show an flg22-induced band-shift (Fig. 5d). In contrast, mutations of other four serine residues did not abolish the flg22-induced band-shift (Supplementary information, Figure S6b). Ser428, Ser435, and Ser436 residues on RGS1 have been reported to be phosphorylated during sugar signaling.^39^ However, simultaneous substitution of these residues with Ala did not impact the flg22-induced band-shift of RGS1CT (Supplementary information, Figure S6b). We further tested whether other patterns similarly induce Ser431 phosphorylation. Treatment of protoplasts with chitin, elf18 and Pep2 all induced phosphorylation of RGS1CT in a Ser431-dependent manner (Fig. 5d), indicating that Ser431 is a major phospho-site induced upon perception of multiple patterns.

BAK1 and PEPR1 kinase domains have been reported to phosphorylate RGS1 at Ser428, but do not appear to phosphorylate Ser431 residue on RGS1.^29,30^ We sought to determine whether BIK1 and related PBL kinases are involved. Because BIK1/PBL family kinases act redundantly in pattern-triggered immunity, we first asked whether flg22-induced RGS1 phosphorylation at Ser431 can be inhibited by AvrAC, a *Xanthomonas campestris campestris* uridylyl transferase known to inhibit multiple BIK1 and PBL kinases.^40^ Expression of AvrAC in protoplasts prevented flg22- and chitin-induced phosphorylation of RGS1 at Ser431 (Fig. 5e), suggesting that BIK1/PBL kinases are indeed involved in phosphorylating RGS1. We recently constructed a series of higher order mutants for RLCK VII coding genes (Rao et al., in submission). Among these, the *rlck vii-4* mutant (*pbl19 pbl20 pbl37 pbl38 pbl39 pbl40*) carried T-DNA insertions in all six genes belonging to clade 4 of RLCK VII. This mutant is severely impaired in chitin signaling but normal in flg22 signaling. Chitin-induced Ser431-dependent RGS1CT phosphorylation was severely diminished in *rlck vii-4* mutant protoplasts, whereas the flg22-induced phosphorylation was normal (Fig. 5f). We previously showed that BIK1 and PBL1, both belonging to clade 8 of RLCKVII, play a key role in Pep-triggered immunity.^41^ The *rlck vii-8* quadruple mutant (*bik1 pbl1 pbl9 pbl11*) carries T-DNA insertions in four genes including *BIK1* and *PBL1* (Rao, in submission). The Pep2-induced Ser431-dependent RGS1CT phosphorylation was impaired in *rlck vii-8* mutant protoplasts (Fig. 5g). Together, these results demonstrate that BIK1/PBL kinases collectively play an essential role in flg22, elf18, chitin, and Pep2-induced phosphorylation of RGS1 Ser431.

We next performed in vitro kinase assay to determine whether BIK1 can phosphorylate RGS1 Ser431. The RGS1CT-FLAG protein was incubated with BIK1^K105E^ (kinase dead) and WT BIK1 in kinase assays. We noticed a protein band shift when RGS1CT-FLAG was incubated with HIS-BIK1 (Fig. 5h). Further study showed that PBL1, the closest homolog of BIK1, also induced RGS1CT-FLAG band shift. (Supplementary information, Figure S6c). However, this band shift could not be induced by BAK1. To verify sites phosphorylated by BIK1 and PBL1, we co-expressed RGS1CT-HIS with GST-tagged BIK1, BIK1^K105E^, and PBL1 in *E. coli*, RGS1CT-HIS protein was purified and subjected to mass spectrometric analyses. While no phospho-peptides were found from RGS1CT co-expressed with BIK1^K105E^, multiple phospho-peptides containing many phospho-serine residues were identified from RGS1CT co-expressed with BIK1 and PBL1 (Supplementary information, Table S2). Among these sites, Ser431 was again identified, indicating RGS1 Ser431 is directly phosphorylated by BIK1. We therefore conclude that BIK1/PBL kinases are responsible for the phosphorylation of RGS1 at Ser431.

### Phosphorylation of Ser431 is required for flg22-triggered RGS1–FLS2 dissociation and de-repression of Gαβγ

**Fig. 6:**
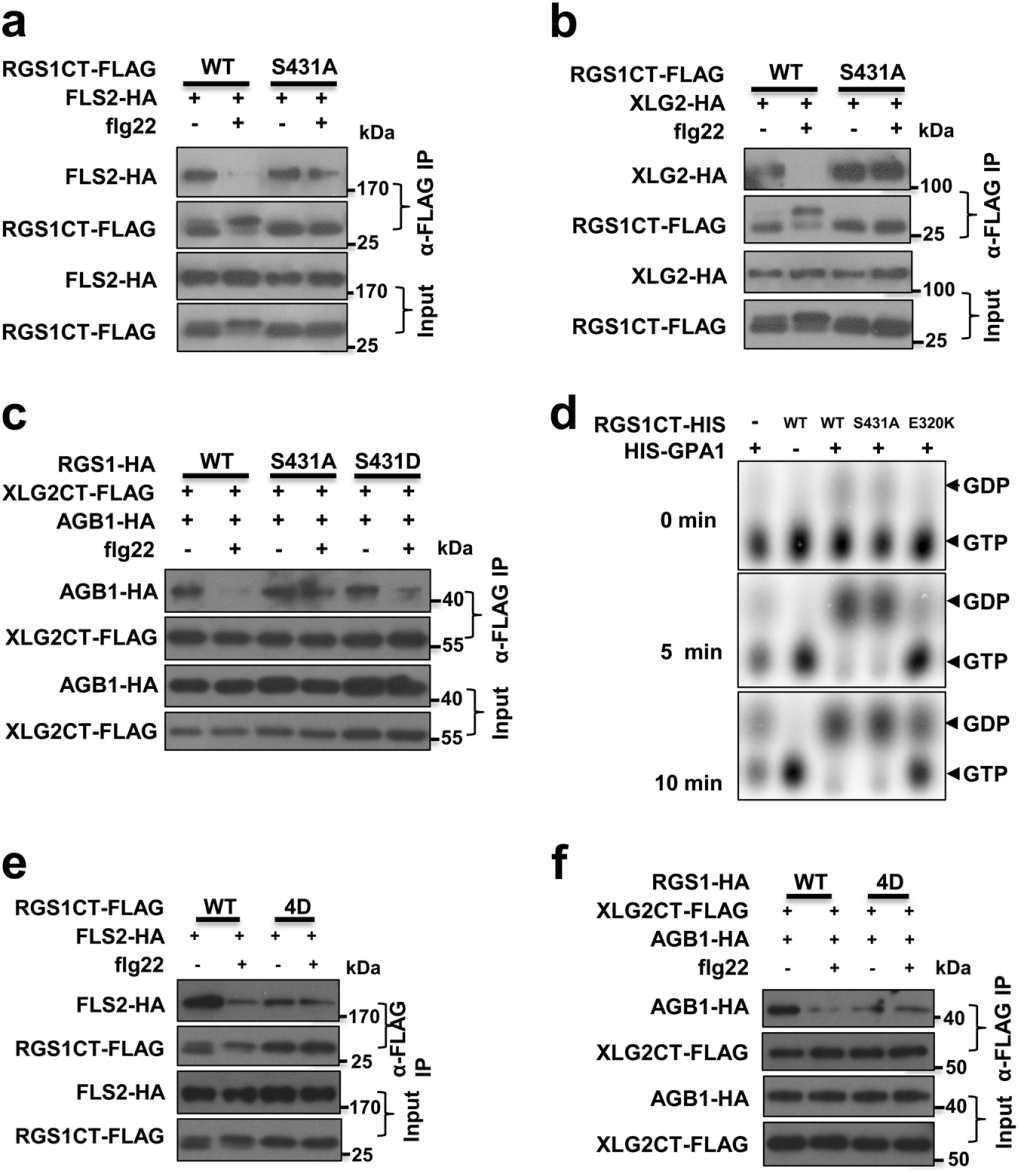
Phosphorylation on RGS1 Ser431 is required for flg22-triggered de-repression of immune signaling. **a** Ser431 phosphorylation is required for dynamic regulation of RGS1CT–FLS2 interaction by flg22. WT protoplasts expressing the indicated constructs were treated with flg22, and co-IP was performed to determine RGS1CT–FLS2 interaction. **b** Ser431 phosphorylation is required for dynamic regulation of RGS1CT–XLG2 interaction by flg22. WT protoplasts expressing the indicated constructs were treated with flg22, and co-IP was performed to determine RGS1CT–XLG2 interaction. **c** RGS1 Ser431 phosphorylation is required for flg22-induced XLG2CT–AGB1 dissociation. XLG2CT-FLAG and AGB1-HA were co-expressed in protoplasts along with RGS1-HA or RGS1^S431A^-HA, treated with or without flg22, and XLG2CT–AGB1 interaction was detected by co-IP assays. **d** The GTPase accelerating activity of RGS1 is not affected by Ser431 mutation. HIS-GPA1 protein was incubated with RGS1CT-HIS or RGS1^S431A^-HIS protein in reaction buffer containing [α^32^P]GTP. The hydrolyzation of GTP was detected by autoradiograph. **e** Phospho-mimicking mutations in Ser428/431/435/436 is sufficient to trigger RGS1 dissociation from FLS2. WT protoplasts expressing the indicated constructs were treated with flg22, and co-IP was performed to detect RGS1CT–FLS2 interaction. **f** Phospho-mimicking mutations in Ser428/431/435/436 is sufficient to trigger XLG2–AGB1 dissociation. XLG2CT-FLAG and AGB1-HA were co-expressed in protoplasts along with RGS1-HA or RGS1^4D^-HA (S428D, S431D, S435D, and S436D). Protoplasts were then treated with or without flg22, and XLG2CT–AGB1 interaction was detected by co-IP assays. The experiments were performed two (**b**, **e**, **f**) or three (**a**, **c**, **d**) times with similar results

We next tested whether RGS1 phosphorylation on Ser431 plays a role in its dynamic interactions with FLS2 and XLG2. Co-IP assays showed that RGS1CT^S431A^ did not disassociate from FLS2 (Fig. 6a) and XLG2 (Fig. 6b) upon flg22 treatment. Luciferase complementation assays showed that RGS1^S431A^ interacted with XLG2 more strongly than did the WT RGS1 (Supplementary information, Figure S7a). These results indicate that Ser431 phosphorylation in RGS1 negatively regulate its interactions with XLG2 and FLS2 and suggest that Ser431 phosphorylation mediates the dynamic regulation of RGS1 by flg22. To further test this possibility, we found by co-IP assays flg22-induced dissociation between XLG2CT and AGB1 in the presence of WT RGS1, RGS1^S431A^, and RGS1^S431D^. In protoplasts expressing WT RGS1, the XLG2CT–AGB1 interaction was readily detected in the absence of flg22 treatment but nearly abolished after flg22 treatment (Fig. 6c), indicating a flg22-induced dissociation of XLG2 and AGB1. In protoplasts expressing the phosphor-dead RGS1^S431A^, the XLG2CT–AGB1 interaction was similarly observed in the presence or absence of flg22 (Fig. 6c), indicating that Ser431 phosphorylation is essential for the flg22-induced XLG2–AGB1 dissociation.

We further tested the possibility that Ser431 may impact the GAP activity. In vitro GTPase assay showed that the GPA1 GTPase activity was accelerated similarly by WT RGS1CT and RGS1CT^S431A^ proteins (Fig. 6d), indicating that the mutation did not affect the GAP activity. We therefore conclude that Ser431 phosphorylation is essential for G protein activation through its dissociation from the FLS2-G protein complex instead of an altered GAP activity.

Protoplasts expressing RGS1^S431D^ showed near normal XLG2CT–AGB1 interaction (Fig. 6c), suggesting that phosphorylation of Ser431 was not sufficient to trigger XLG2–AGB1 dissociation. We reasoned that additional phospho-sites may be required. Because Ser428 was also phosphorylated by BIK1 in vitro (Supplementary information, Table S2) and Ser428/435/436 were previously reported to be required for flg22-induced endocytosis of RGS1,^29^ we tested whether phosphorylation of these residues in addition to Ser431 can trigger RGS1 dissociation from FLS2 and G protein activation. We simultaneously mutated Ser428/431/435/Ser436 to phospho-mimicking aspartic acid and constructed the RGS1CT^4D^-FLAG and RGS1^4D^-HA constructs. Co-IP assays showed that RGS1CT^4D^-FLAG was impaired in its interaction with FLS2, and the interaction was insensitive to flg22 treatment (Fig. 6e). Co-IP assays additionally showed that XLG2CT–AGB1 and GPA1–AGB1 interactions were greatly impaired in protoplasts expressing RGS1^4D^-HA compared to that expressing WT RGS1 (Fig. 6e; Supplementary information, Figure S7b). These results strongly support that phosphorylation of these four residues are sufficient to trigger RGS1 dissociation from FLS2 and activation of Gαβγ.

### Phosphorylation of RGS1 Ser431 is required for flg22-triggered immune signaling

**Fig. 7:**
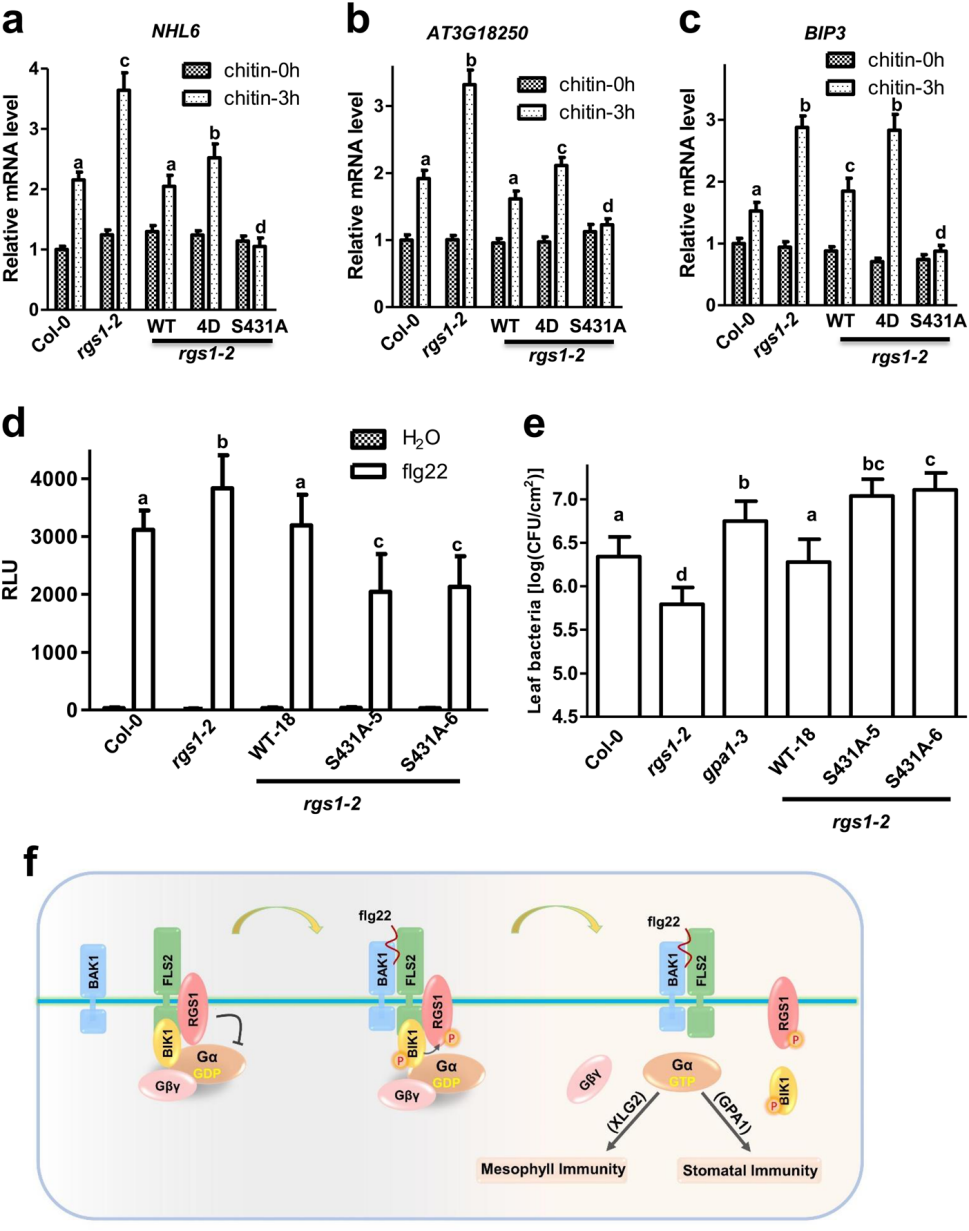
Phosphorylation of RGS1 positively regulates immune signaling. **a**–**c** Mutations of RGS1 phosphosites modulate chitin-induced defense gene expression. Protoplasts from *rgs1-2* plants were transfected with different forms of *RGS1-HA* plasmids, treated with chitin for 3 h, and qPCR was performed for the expression of *NHL6* (**a**), *AT3G18250* (**b**), and *BIP3* (**c**). Different letters indicate significant difference at *P* < 0.05 (mean ± SD, one-way ANOVA followed by Tukey’s post hoc test). **d** RGS1^S431A^ confers increased inhibition of immune signaling. *rgs1-2* was complemented with the WT *RGS1* (WT) or *RGS1*^*S431A*^ mutant (S431A) constructs, and resulting lines were examined for flg22-triggered H_2_O_2_ production. Different letters indicate significant difference at *P* < 0.05 (mean ± SD, *n* ≥ 6, one-way ANOVA followed by Tukey’s post hoc test). **e** RGS1^S431A^ confers increased susceptibility to *Pst* DC300 upon spray inoculation. The indicated genotypes were sprayed with *Pst* DC3000 at 5 × 10^8^ CFU/mL and bacterium number was determined 3 days later. Different letters indicate significant difference at *P* < 0.05 (mean ± SD, *n* ≥ 6, one-way ANOVA followed by Tukey’s post hoc test). **f** Model for RGS1-Gαβγ regulation by FLS2. In the resting state, the Gα^GDP^βγ heterotrimers associate with FLS2, and this is maintained by interactions of RGS1 with FLS2 and XLG2/GPA1. Perception of flg22 induces the formation of an active FLS2-BAK1 receptor complex and BIK1/PBLs-dependent phosphorylation of RGS1 at Ser428/431, which promotes the dissociation of RGS1 from FLS2 and the G proteins. In the absence of RGS1, Gα^GDP^ is spontaneously converted to Gα^GTP^, the latter dissociates from Gβγ to activates its effector proteins such as RbohD. Gα hydrolyzes GTP through its intrinsic GTPase activity and cycles back to Gα^GDP^. The experiments were performed two (**a**–**c**) or three (**d**, **e**) times with similar results

To determine whether the Ser431 phosphorylation is required for the function of RGS1, we first checked chitin-induced defense gene expression. We transiently expressed different forms of RGS1-HA in *rgs1-2* protoplasts and detected the defense gene expression upon chitin treatment. Compared to Col-0 protoplasts, *rgs1-2* mutant protoplasts showed increased expression of *NHL6*, *At3G18250*, and *BIP3*. While WT RGS1 suppressed the marker gene expression to the Col-0 levels, the S431A variant rendered the marker gene expression completely insensitive to chitin treatment (Fig. 7a–c). Consistent with the reduced stability of XLG2-Gβγ heterotrimer, *rgs1-2* protoplasts expressing RGS1^4D^ displayed stronger chitin-induced expression of marker genes compared to protoplasts expressing WT RGS1 (Fig. 7a–c). These findings suggested that chitin-induced phosphorylation of Ser431, and likely Ser428/435/Ser436 as well, leads to de-repression of Gαβγ.

To further test the role of RGS1 Ser431 phosphorylation in immune signaling, we complemented *rgs1-2* mutant plants with the S431A variant of *RGS1-FLAG-GFP* transgene under control of the native *RGS1* promoter and tested independent T2 lines for flg22-induced ROS production. As expected, the transgenic line carrying the WT *RGS1-FLAG-GFP* transgene showed significantly less ROS production compared to *rgs1-2* and was indistinguishable from Col-0 plants (Fig. 7d). Although the two transgenic lines carrying a phospho-dead *RGS1*^*S431A*^*-FLAG-GFP* variant accumulated much less RGS1 protein than did the WT *RGS1-FLAG-GFP* line (Supplementary information, Figure S8), they were significantly reduced in ROS production compared to the WT *RGS1-FLAG-GFP* line, indicating that the RGS1^S431A^ variant suppressed immune signaling more strongly than did the WT form. We further spray-inoculated these lines with *Pst* and measured bacterial growth in plants. As predicted, the *rgs1-2* and *gpa1-3* plants showed elevated resistance and increased susceptibility, respectively (Fig. 7e). The WT *RGS1-FLAG-GFP* transgene fully complemented the *rgs1* mutation and the transgenic plants supported normal growth of *Pst* indistinguishable from Col-0 plants. In contrast, the *RGS1*^*S431A*^*-FLAG-GFP* lines were highly susceptible to *Pst* and supported even greater amounts of bacterial growth than did *gpa1* plants. The inhibitory effect on XLG2 in the *RGS1*^*S431A*^*-FLAG-GFP* lines may account for the greater susceptibility than *gpa1* plants. Thus RGS1^S431A^ is a gain-of-function mutation with stronger inhibition of immunity.

## Discussion

In this study, we show that the flg22-induced dissociation of Gα from Gβγ correlates with guanine nucleotide-binding states and is required for immune signaling. RGS1 directly interacts with FLS2, and is required for stability of Gαβγ in the FLS2 receptor complex. Consistent with this, we show that RGS1 plays a negative role in flg22- and chitin-triggered immune signaling and disease resistance to *Pst*. Most importantly, we show that the perception of flg22 triggers a BIK1/PBL-mediated phosphorylation on RGS1, which is required for the flg22-induced dissociation of RGS1 from XLG2 and FLS2 and necessary for FLS2-mediated immune signaling, suggesting that this phosphorylation triggers ligand-dependent de-repression of the G proteins.

Although XLG2 is known to bind both GDP and GTP and possesses GTPase activity,^12^ it remains unknown whether GDP- and GTP-bindings regulate XLG2-Gβγ stability and the XLG2-mediated immune signaling. We show that application of GDP enhances the XLG2–AGB1 interaction, whereas addition of GTP-γ-S reduces the interaction. These results are indicative of increased and decreased stability of XLG2-Gβγ upon GDP and GTP binding, respectively. Disruption of guanine nucleotide-binding motifs strongly enhanced XLG2–AGB1 and XLG2–FLS2 interactions, further supporting that guanine nucleotide-binding controls XLG2-Gβγ activation in the FLS2 receptor complex. Disruption of these motifs in XLG2 significantly impaired the flg22-triggered ROS production independent of BIK1 stability, indicating that the guanine nucleotide-binding is required for downstream immune signaling.

RGS1 is known to negatively regulate heterotrimeric G protein signaling in *Arabidopsis*.^5,6,8,34,36,37^ The role of RGS1 in regulating immune signaling has been controversial. Two previous studies suggested a positive role of RGS1 in flg22-induced ROS production.^32,33^ However, RGS1 is a negative regulator of Gα, and both GPA1 and XLG2 are known to positively mediate immune signaling downstream of FLS2.^11,24–28^ We show convincing evidence that RGS1 negatively regulates flg22- and chitin-triggered signaling. The flg22- and chitin-induced ROS production and defense gene expression, disease resistance to *Pst*, and stomatal defense were all elevated in *rgs1* mutants.

The XLG2–AGB1 interaction in the absence of flg22 treatment suggests an inactive XLG2-Gβγ heterotrimer in the resting state. This questions how XLG2-Gβγ is maintained in an inactive state, as plant Gα proteins can bind GTP in the absence of GEFs and are thus self-activating. Our analyses show that RGS1 plays a decisive role in maintaining the inactive XLG2-Gβγ in the FLS2 receptor complex. RGS1 negatively regulates FLS2-mediated immunity upstream of XLG2/3. In the absence of flg22 treatment, RGS1 directly interacts with FLS2 kinase domain and XLG2 C-terminus to stabilize XLG2–AGB1 and XLG2–FLS2 interactions. These indicate that, in the resting state, RGS1 maintains XLG2-Gβγ in the FLS2 receptor complex. The RGS1 mutant lacking the GAP activity is completely unable to stabilize the XLG2–AGB1 interaction. Furthermore, disruption of guanine nucleotide-binding motifs renders the XLG2–AGB1 interaction insensitive to the *rgs1-2* mutation. Together these results strongly support that RGS1 stabilizes XLG2-Gβγ trimer in the FLS2 complex by accelerating the GTP hydrolysis in XLG2, explaining how a self-activating plant Gα protein is maintained in an inactive state prior to receptor activation.

Most importantly, we show how RGS1 mediates flg22-triggered activation of Gαβγ. Flg22 treatment induces phosphorylation of RGS1 and its dissociation from both XLG2 and FLS2. RGS1 has been shown to be phosphorylated at Ser428/435/436 to promote its endocytosis during sugar signaling.^39^ These sites have been also shown to be required for flg22-induced endocytosis of RGS1.^29^ It remains unknown, however, whether these residues are required for G protein activation and immune signaling. We show that Ser431 is a major phosphosite upon treatments by flg22, elf18, chitin and Pep2. Unlike WT RGS1, RGS1^S431A^ prevents flg22-induced XLG2-Gβγ activation. In plants, RGS1^S431A^ inhibits immune responses more strongly than does the WT RGS1. The increased RGS1CT–XLG2 interaction and stronger immune suppression conferred by the S431A substitution indicate that the protein is a gain-of-function mutant but not an inactive protein. In soybean, the NFR1-mediated phosphorylation of RGS enhances its GAP activity and nodulation.^8^ The RGS1^S431A^ mutation, however, does not impact its GAP activity. Instead, RGS1^S431A^ is impaired in dissociation from XLG2 and FLS2 in response to flg22, indicating that Ser431 phosphorylation regulates Gαβγ activation through its dissociation from the FLS2-G protein complex instead of altered GAP activity.

Phospho-mimicking mutations in Ser428/431/435/436, but not in Ser431 alone, are sufficient for triggering RGS1–FLS2 dissociation and Gαβγ activation. These results suggest that, while phosphorylation of Ser431 plays a major role in FLS2-mediated regulation of G proteins, simultaneous phosphorylation of Ser428/431/435/436 is necessary to mediate the flg22-induced RGS1–FLS2 dissociation and G protein activation. In support of this, Ser428 is phosphorylated by BAK1 and PEPR1 in vitro, and its phosphorylation is enhanced by flg22 treatment.^29^ These results suggest that phosphorylation of RGS1 at Ser428/431/435/436 collectively serves as a molecular switch to activate XLG2-dependent immune signaling upon perception of flg22.

Inhibition of Ser431 phosphorylation by AvrAC and our higher order mutant analyses demonstrate that BIK1/PBL kinases are collectively required for RGS1 phosphorylation at Ser431. In vitro kinase assay showed that BIK1 and PBL1 can phosphorylate RGS1. Mass spectrum analyses identified many phosphos-sites including Ser428 and Ser431 when RGS1CT was co-expressed with BIK1 in *E. coli*, confirming that BIK1/PBLs can phosphorylate RGS1 at Ser428 and Ser431.

We previously showed that perception of flg22 also leads to phosphorylation of XLG2 N-terminus by BIK1, which is necessary for full function of XLG2-mediated immune signaling.^11^ RGS1 interacts with XLG2CT and stabilizes its interaction with AGB1, suggesting that the N-terminus of XLG2 is not required for the RGS1-mediated regulation (Fig. 3f). Thus, the phosphorylation on N-terminal domain of XLG2 may additionally regulate immune responses through an unknown mechanism, which is beyond the scope of the current study.

The mechanism discussed above also applies to the control of GPA1 and PRRs other than FLS2. The *rgs1* mutants display reduced stomatal aperture that is insensitive to *Pst* *hrcC*^*-*^ treatment. The finding is consistent with a role of GPA1 in flg22-induced stomatal closure, and thus supports that RGS1 regulates GPA1 downstream of FLS2. Expression of RGS1^4D^ in protoplasts impairs GPA1–AGB1 interaction. The RGS1^S431A^ mutant conferred increased disease susceptibility to *Pst* when spray-inoculated, suggesting an inability to activate GPA1 when RGS1 Ser431 phosphorylation is impaired. Furthermore, Ser431 is phosphorylated upon induction by multiple patterns, and *rgs1* mutants display elevated defense gene expression and ROS production in response to chitin. This notion is further supported by sensitized defense gene induction by chitin in protoplasts expressing RGS1^4D^ variant compared to that expressing WT RGS1. These results suggest that RGS1 and XLG2 are also involved in the regulation of immune signaling downstream of PRRs other than FLS2.

Loss-of-function *rgs1* mutations lead to G protein activation, but not constitutive ROS production. This is expected, since RBOHD is subjected to multiple layers of regulation including phosphorylation by BIK1/PBL1 and CPK5, and calcium-binding, which are all essential for RBOHD activation.^42^ The activated XLG2 is not sufficient for RBOHD activation. Instead, it only serves to enhance its activity. Note that in *RGS1*^*S431A*^*-FLAG-GFP* and *XLG2ΔG* transgenic lines, XLG2 is expected to remain in the resting state. However, the flg22-induced ROS in these lines is only partially impaired and is still greater than that in *xlg2* plants. These results are completely consistent with our previous findings that, in the resting state, XLG2-Gβγ stabilizes BIK1.^11,43^ The greater amount of BIK1 accounts for greater ROS production in *XLG2ΔG* transgenic lines than in *xlg2 xlg3* plants upon flg22 treatment. Thus, XLG2 regulates ROS at two levels. In the resting state, XLG2-Gβγ stabilizes BIK1 independent of GTP-binding, which positively regulate flg22-induced ROS. After activation, the GTP-bound XLG2 further regulates RBOHD through an unknown mechanism.^11^ This and our previous studies additionally uncover a GTP-independent function of a Gα protein, which has not been reported previously.^11,43^

A major dilemma in heterotrimeric G protein signaling is how the self-activating plant G proteins respond to extracellular signals. Our study uncovers a mechanism through which a single-transmembrane receptor regulates heterotrimeric G proteins in plants (Fig. 7f). In the resting state, RGS1 directly interacts with the FLS2 receptor complex and maintains Gα in a GDP-bound state, which involves accelerated hydrolysis of GTP. This arrests the Gαβγ heterotrimer in the FLS2 receptor complex. Upon activation by flg22, RGS1 is phosphorylated in the C-terminal tail at multiple sites. Among these, Ser431 is the primary site phosphorylated by BIK1/PBLs and is essential for flg22-triggered G protein activation. This phosphorylation triggers RGS1 dissociation from FLS2 and Gα. In the absence of RGS1-mediated repression, the self-activating Gα proteins spontaneously exchange GDP for GTP, which then dissociates from Gβγ dimer and the FLS2 receptor to regulate downstream signaling components. The two Gα proteins XLG2 and GPA1 regulate distinct aspects of immune responses. While GPA1 controls stomatal closure, XLG2 enhances flg22 and chitin-triggered ROS production and defense gene expression. The coupling to a receptor kinase and ligand-induced de-repression of Gαβγ contrast the coupling to GPCRs and ligand-induced activation of animal/fungal heterotrimeric G proteins. As GPA1 is known to act in numerous biological processes, and plant heterotrimeric G proteins appear to be commonly coupled to RKs or RLPs,^7–10,44–46^ the mechanism depicted in this study has broad implications. Future work will test whether plant RGS proteins arrest the heterotrimeric G proteins in other receptor complexes and whether ligand perception relieves the RGS-mediated repression on Gα proteins to regulate diverse processes in plants.

## Materials and methods

### *A. thaliana* and *N. benthamiana*

*Arabidopsis* plants used in this study include WT (Col-0), *rgs1-1* and *rgs1-2*,^5^ *xlg2 xlg3*,^47^ *rlck vii-8* (*bik1 pbl1 pbl9 pbl11*),* rlck vii-4* (*pbl19 pbl20 pbl37 pbl38 pbl39 pbl40*), and *BIK1-HA* transgene.^21^ Plants were grown in soil at 22 °C with 10/14 h day/night photoperiod or 1/2 MS medium containing 1% sucrose.

Leaves of four- to five-week-old soil-grown *Arabidopsis* plants were used for protoplast preparation, bacterial infection, stomatal aperture measurements and flg22 and chitin-induced ROS production assays. *N. benthamiana* plants used for luciferase-complementation assay were soil-grown under a 10/14 h photoperiod at 22 °C.

### Bacterial strains and culture conditions

*E. coli* was grown on LB plates or liquid media with appropriate antibiotics at 37 °C. Strains of *Agrobacterium* (GV3101) were grown on LB plates or liquid media with appropriate antibiotics at 28 °C. *Pst* DC3000 and *Pst* DC3000 *hrcC*^*−*^ strains were grown on KB plates or liquid media containing 100 mg/mL rifampicin at 28 °C.

### Constructs

Constructs and transgenic plants related to XLG2 have been described previously.^11^ The GPA1 coding sequence was PCR-amplified and inserted into pUC19-35S-FLAG-RBS to generate the GPA1-FLAG construct. To generate *RGS1* transgenic lines in *rgs1-2* plants, the RGS1 coding sequence driven by a native promoter was fused with the C-terminus FLAG-GFP tag followed by the RBS terminator and cloned into pCAMBIA 1300 vector. The constructs were introduced into *rgs1-2* plants by *Agrobacterium*-mediated transformation. To generate full-length and truncated RGS1-FLAG/HA constructs for protoplasts transfection, the coding sequence of RGS1 or C-terminal region of RGS1 (RGS1CT, 249-459) was PCR-amplified and cloned into pUC19-35S-FLAG/HA-RBS vector. For HIS and GST fusion constructs, the RGS1CT coding sequence was inserted into pET28a and pGEX 6P-1 vectors. To generate constructs for luciferase complementation assays, the corresponding genes were amplified from cDNA and cloned into pCAMBIA1300-35S-Cluc-RBS or pCAMBIA1300-35S-HA-Nluc-RBS vector. Mutant constructs were generated by site-directed mutagenesis using the WT constructs as templates. The primers used in this study were listed in the supplementary information (Supplementary information, Table S1).

### Bacterial growth assay

Leaves of four–five-week-old soil grown plants were infiltrated with *Pst* DC3000 strain at 1 × 10^6^ CFU/mL or sprayed at 5 × 10^8^ CFU/mL. Leaf bacterial number was determined 3 days post inoculation.

### Oxidative burst measurement

Leaves of four-week-old plants were sliced into 1 mm strips and incubated in water for 12 h in 96-well plates. The leaf strips were treated with luminescence detection buffer (20 μM luminol and 10 μg/mL horseradish peroxidase) containing 1 μM flg22 or 200 μg/mL chitin and luminescence was recorded by GLOMAX 96 microplate luminometer (Promega) as describe.^48^ Each treatment includes leaves from at least 6 individual plants of each material.

### Co-IP assay

Protoplasts isolated from soil-grown plants were transfected with desired plasmids, incubated overnight, and treated with 1 μM flg22, elf18, or Pep2 or 200 μg/mL chitin for 5 min. Total protein was extracted with protein extraction buffer (50 mM HEPES [pH 7.5], 150 mM KCl, 10 mM EDTA, 0.5% Trition-X 100, 1 mM DTT, proteinase inhibitor cocktail) and incubated with anti-FLAG M2 agrose (Sigma) for 30 min, washed with extraction buffer for 4 times and eluted with 3 × FLAG peptide (Sigma). Different guanine nucleotides at 100 μM concentration were added to the protein extraction buffer as desired. To determine effects of different RGS1 variants on XLG2/GPA1–Gβγ interactions, *Arabidopsis* protoplasts were transfected with the following constructs: 100 μg XLG2-FLAG or GPA1-FLAG, 100 μg AGB1-HA, and 5–10 μg RGS1-HA. Total protein was extracted with protein extraction buffer and incubated with anti-FLAG M2 agrose (Sigma) for 20 min. The immunoprecipitates were separated by SDS-PAGE and protein interactions were detected with anti-HA and anti-FLAG immunoblots.

### GST pull-down assay

The recombinant proteins were expressed in *E. coli* and  purified using the glutathione agarose beads (for GST-tagged proteins) or Ni-NTA agarose beads (for HIS-tagged proteins). For GST pull-down assay, 5 μg GST- and HIS-tagged proteins were incubated with 30 μl glutathione agarose beads in 100 μl GST buffer (25 mM Tris-HCl, 100 mM NaCl, 1 mM DTT, pH 7.5) for 1 h at 4 °C, washed 5 times with GST wash buffer (25 mM Tris-HCl, 300 mM NaCl, 1 mM DTT, pH 7.5), and eluted with GST buffer containing 15 mM GSH. The presence of HIS-tagged proteins was examined by anti-HIS immunoblot.

### Luciferase complementation assay

The luciferase complementation assay was performed as previously described.^49^ Nluc and Cluc constructs were co-expressed in *N. benthamiana* leaves by *Agrobacterium-*mediated transient expression. Leaf disks were taken 2 days later, incubated with 1 mM luciferin in a 96-well, and the luc activity was measured by the GLOMAX 96 microplate luminometer.

### RNA isolation and qPCR

Four-to-five-week-old soil-grown *Arabidopsis* plants were infiltrated with 1 μM flg22 or 200 μg/mL chitin for 3 h, and total RNA was extracted using the RNeasy Plant Mini Kit (Qiagen) by following the manufacturer’s instructions. To extract the RNA from protoplasts, protoplasts were transfected with desired constructs, incubated overnight, treated with chitin for 3 h, and total RNA was extracted with the RNeasy Plant Mini Kit (Qiagen). cDNA was synthesized with the SuperScriptIII First-Strand Kit (Invitrogen) and used for qPCR analysis. qPCR was performed by using cDNA template and ACT8 was used as a control gene.

### Phospho-site identification

To identify the phospho-sites in RGS1, WT protoplasts expressing RGS1CT-FLAG was treated with 1 μM flg22 for 5 min, lysed in protein IP buffer (50 mM HEPES [pH 7.5], 150 mM KCl, 1 mM EDTA, 0.5% Trition-X 100, 1 mM DTT, proteinase inhibitor cocktail), and the RGS1CT-FLAG protein was purified using anti-FLAG M2 agarose (Sigma). The immunoprecipitates were separated in 10% NuPAGE gel (Invitrogen) and subject to LC-MS/MS analysis as previously described.^23^

To identify BIK1- and PBL1-phosphorylated sites in RGS1, the GST-tagged BIK1, PBL1, or BIK1^K105E^ was co-expressed with HIS-tagged RGS1CT in *E. coli* and purified using Ni-NTA agarose beads. RGS1CT-HIS protein was separated in 10% NuPAGE gel (Invitrogen) and subjected to LC-MS/MS analysis.

For in vitro phosphorylation of RGS1 isolated from protoplasts, *Arabidopsis* protoplasts were transfected with 100 μg *RGS1CT-FLAG* plasmid. RGS1CT-FLAG was affinity-purified, incubated with 200 ng HIS-BIK1 in the kinase reaction buffer (25 mM Tris-HCl, 10 mM MgCl 2, 1 mM DTT, 100 mM ATP, pH 7.5) for 30 min at 30 °C, and phosphorylation of RGS1 (band-shift) was detected by immunoblot analyses.

### Stomatal aperture measurements

Four-to-five -week-old *Arabidopsis* plants were kept under light for 3 h to ensure the stomata were open prior to treatment. Epidermal strips were peeled from the rosette leaves and floated in water or a suspension containing 5 × 10^8^ CFU/mL *Pst* DC300 *hrcC*^*−*^ for 2 h before being examined as previously described.^50^

### GTPase activity assay

GTP hydrolysis assays were performed as described.^12,51^ HIS-tagged GPA1 and different variants of RGS1CT were purified from *E. coli*. 0.1 μg HIS-GPA1 was incubated with or without 0.02 μg RGS1CT-HIS in the reaction buffer (Tris pH 8.0, 50 mM; MgCl_2_, 10 mM; EDTA, 1 mM; DTT, 1 mM) containg [α^32^P]GTP (5 μCi) at 30 °C. Samples were withdrawn at the indicated time points, and the reaction was stopped by addition of an equal volume 0.5 M EDTA (pH 8.0). Samples were spotted onto PEI-cellulose TLC plates (Sigma), developed in 0.5 M KH_2_PO_4_ (pH 3.4) solution, and detected by autoradiograph.

## Electronic supplementary material

Supplementary figure S1(PDF 215 kb)

Supplementary figure S2(PDF 280 kb)

Supplementary figure S3(PDF 201 kb)

Supplementary figure S4(PDF 123 kb)

Supplementary figure S5(PDF 120 kb)

Supplementary figure S6(PDF 199 kb)

Supplementary figure S7(PDF 194 kb)

Supplementary figure S8(PDF 99 kb)

Supplementary table S1(PDF 102 kb)

Supplementary table S2(PDF 116 kb)
