# Supplementary material for: Ligand-triggered de-repression of Arabidopsis heterotrimeric G proteins coupled to immune receptor kinases
Source: Cell Res. 2018 Mar 15;28(5):529–43. doi: 10.1038/s41422-018-0027-5 (PMC5951851; doi:10.1038/s41422-018-0027-5)
Supplement: Supplementary file 1 — Supplementary figure S1(PDF 215 kb) [file 41422_2018_27_MOESM1_ESM.pdf]

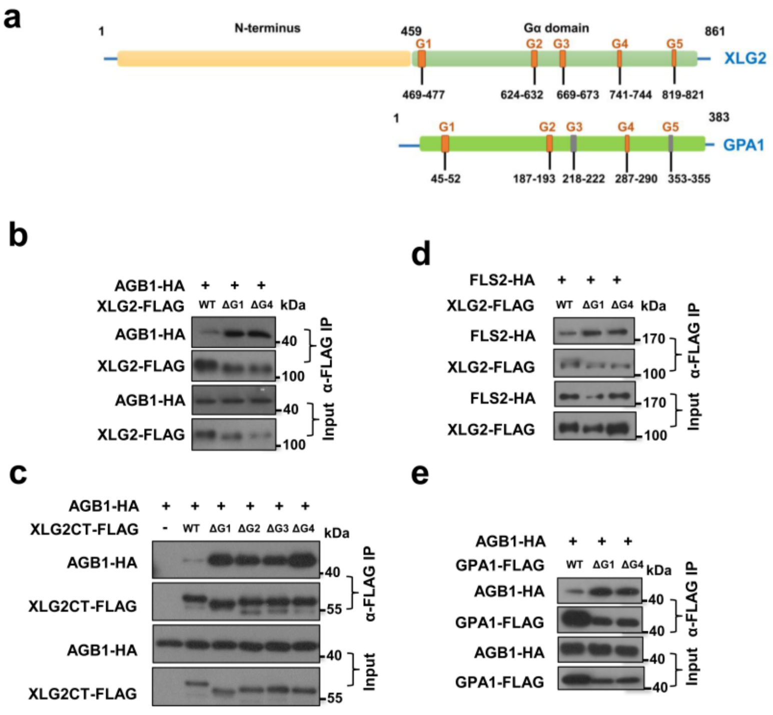


**Supplementary information, Figure S1. Disruption of guanine nucleotide-binding motifs stabilizes interactions of Gα proteins with AGB1 and BIK1.**

(a) Schematic representation of guanine nucleotide-binding (G) motifs in XLG2 and GPA1. Positions of each motif are indicated below the bar. Note that putative G3 and G5 in XLG2 do not possess residues conserved in canonical Gα. Total number of amino acids in each protein is indicated on top of the bar.

(b) XLG2 mutants defective in guanine nucleotide-binding motifs are stabilized in their interactions with AGB1. The indicated constructs were transfected into Col-0 protoplasts, and co-IP was performed.

(c) Disruption of guanine nucleotide-binding motifs enhances XLG2CT-AGB1 interaction. The indicated constructs were transiently expressed in Col-0 protoplasts for co-IP assays.

(d) XLG2 mutants defective in guanine nucleotide-binding motifs are stabilized in their interactions with FLS2. The indicated constructs were transfected into Col-0 protoplasts, and co-IP was performed.

(e) Disruption of guanine nucleotide-binding motifs enhances GPA1-AGB1 interaction.

The experiments were performed two (a and e) or three (b and d) times with similar results.
