# Supplementary material for: Ligand-triggered de-repression of Arabidopsis heterotrimeric G proteins coupled to immune receptor kinases
Source: Cell Res. 2018 Mar 15;28(5):529–43. doi: 10.1038/s41422-018-0027-5 (PMC5951851; doi:10.1038/s41422-018-0027-5)
Supplement: Supplementary file 2 — Supplementary figure S2(PDF 280 kb) [file 41422_2018_27_MOESM2_ESM.pdf]

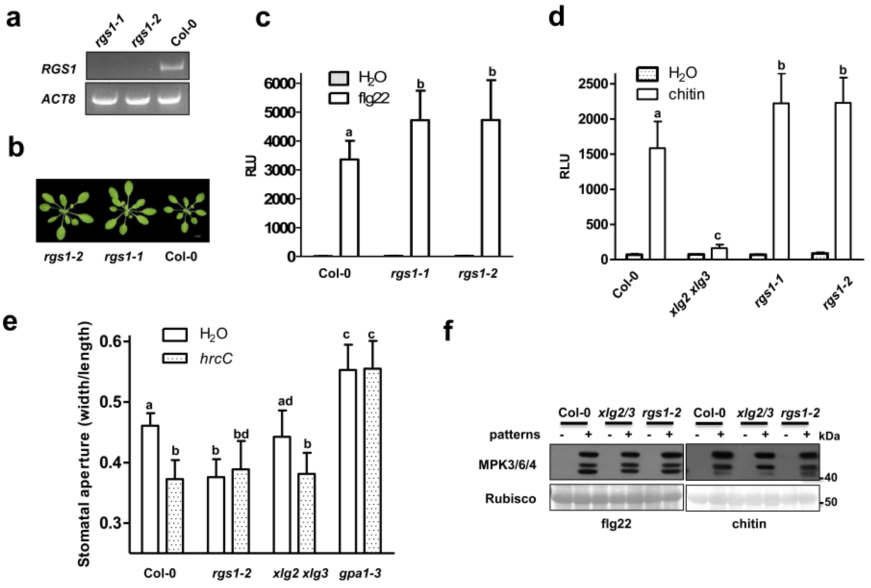

Supplementary information, Figure S2. *rgs1* shows reduced pattern-triggered ROS production and normal MAPK activation.

(a) *RGS1* transcripts were not detected in *rgs1-1* and *rgs1-2* mutants. Seedlings of the indicated genotypes were examined for *RGS1* transcripts using semi-quantitative RT-PCR.

(b) Morphology of 4-week-old *rgs1-1* and *rgs1-2* plants in short-day conditions.

(c) *rgs1-2* and *rgs1-1* display enhanced ROS production in response to flg22. Different letters indicate significant difference at $P<0.05$ (mean ± SD, n ≥6, one-way ANOVA followed by Tukey’s post hoc test).

(d) *rgs1-2* and *rgs1-1* display enhanced ROS production in response to chitin. Different letters indicate significant difference at $P<0.05$ (mean ± SD, n ≥6, one-way ANOVA followed by Tukey’s post hoc test).

(e) *gpa1* and *rgs1*, but not *xlg2 xlg3* display defects in stomatal aperture. Leaf epidermis of the indicated genotypes were treated with or without *Pst hrcC*$^-$ before examined for stomata aperture. Different letters indicate significant difference at $P<0.05$ (mean ± SD, n >30, one-way ANOVA followed by Tukey’s post hoc test).

(f) MPKs are normally activated by patterns in *xlg2 xlg3* and *rgs1-2* seedlings. Seedlings of the indicated genotypes were treated with flg22 and chitin, and MPK activation was determined by anti-pERK immunoblot.

The experiments were performed two (c and d) or three (e and f) times with similar results.
