# Supplementary material for: Ligand-triggered de-repression of Arabidopsis heterotrimeric G proteins coupled to immune receptor kinases
Source: Cell Res. 2018 Mar 15;28(5):529–43. doi: 10.1038/s41422-018-0027-5 (PMC5951851; doi:10.1038/s41422-018-0027-5)
Supplement: Supplementary file 3 — Supplementary figure S3(PDF 201 kb) [file 41422_2018_27_MOESM3_ESM.pdf]

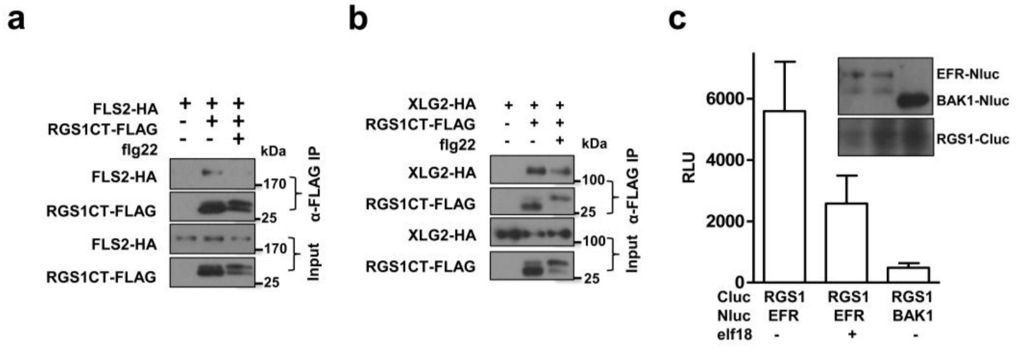

Supplementary information, Figure S3. RGS1 interacts with XLG2, FLS2 and EFR.

(a-b) RGS1CT dynamically interacts with FLS2 and XLG2 in protoplasts. Col-0 protoplasts expressing the indicated constructs were treated with flg22 and subjected to co-IP assays.

(c) RGS1 dynamically interacts with EFR in *Nicotianan benthamiana* as detected by luciferase complementation assays (mean ± SD; n ≥ 6).

The experiments were performed three times with similar results.
