# Supplementary material for: Ligand-triggered de-repression of Arabidopsis heterotrimeric G proteins coupled to immune receptor kinases
Source: Cell Res. 2018 Mar 15;28(5):529–43. doi: 10.1038/s41422-018-0027-5 (PMC5951851; doi:10.1038/s41422-018-0027-5)
Supplement: Supplementary file 4 — Supplementary figure S4(PDF 123 kb) [file 41422_2018_27_MOESM4_ESM.pdf]

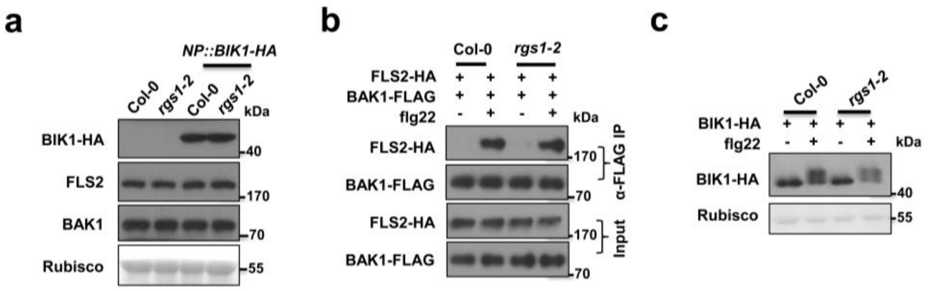

Supplementary information, Figure S4. RGS1 is not required for the formation of an active FLS2 receptor complex nor stability of components in the receptor complex.

(a) *RGS1* is not required for the accumulation of BIK1, FLS2, and BAK1 proteins. Seedlings of the indicated genotypes were examined with immunoblot analyses.

(b) *RGS1* is not required for flg22-induced FLS2-BAK1 dimerization. Col-0 and *rgs1-2* protoplasts were transfected with the indicated constructs, treated with flg22 before co-IP assays were performed.

(c) *RGS1* is not required for flg22-induced BIK1 phosphorylation. Col-0 and *rgs1-2* protoplasts expressing BIK1-HA was treated with flg22 before immunoblot analysis. BIK1 phosphorylation is indicated by an upward band-shift.

All experiments were performed twice with similar results.
