# Supplementary material for: Ligand-triggered de-repression of Arabidopsis heterotrimeric G proteins coupled to immune receptor kinases
Source: Cell Res. 2018 Mar 15;28(5):529–43. doi: 10.1038/s41422-018-0027-5 (PMC5951851; doi:10.1038/s41422-018-0027-5)
Supplement: Supplementary file 5 — Supplementary figure S5(PDF 120 kb) [file 41422_2018_27_MOESM5_ESM.pdf]

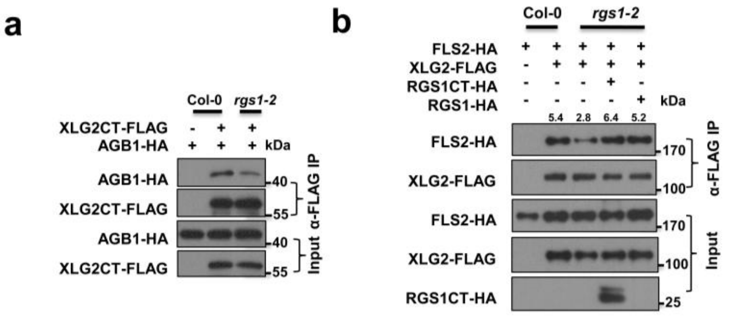

Supplementary information, Figure S5. RGS1 is required for the stability of FLS2-G protein complex.

(a) *RGS1* is required for stable XLG2CT-AGB1 interaction. The indicated constructs were transiently expressed in Col-0 or *rgs1-2* mutant protoplasts and subjected to co-IP assays.

(b) *RGS1* is required for stability of XLG2-FLS2 interactions.

The experiments were performed twice with similar results.
