# Supplementary material for: Ligand-triggered de-repression of Arabidopsis heterotrimeric G proteins coupled to immune receptor kinases
Source: Cell Res. 2018 Mar 15;28(5):529–43. doi: 10.1038/s41422-018-0027-5 (PMC5951851; doi:10.1038/s41422-018-0027-5)
Supplement: Supplementary file 6 — Supplementary figure S6(PDF 199 kb) [file 41422_2018_27_MOESM6_ESM.pdf]

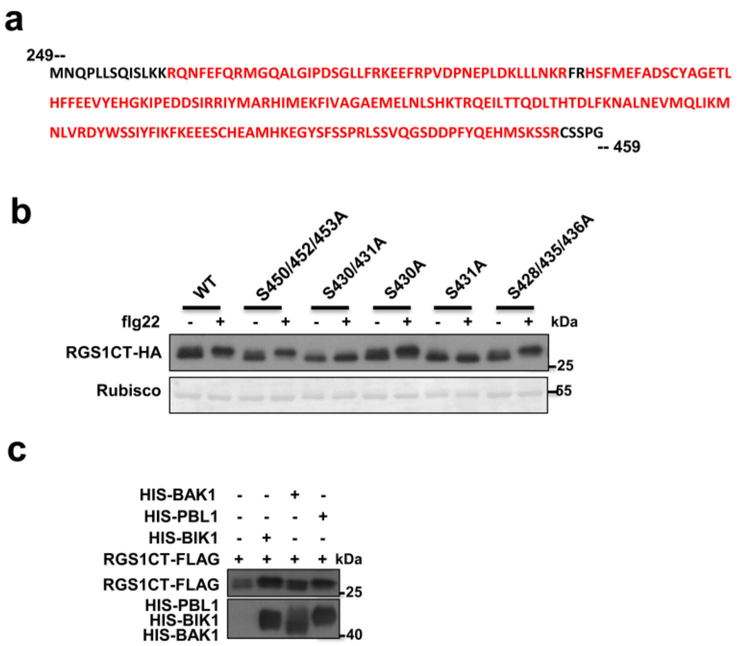

Supplementary information, Figure S6. Flg22 induces RGS1 phosphorylation at Ser431.

(a) Peptide coverage of RGS1CT in LC-MS/MS. RGS1CT-FLAG was affinity purified from protoplasts following flg22 treatment and subjected to mass spectrometry analysis. Amino acid sequence covered by peptides identified in LC-MS/MS is indicated in red.

(b) Ser428, Ser435 and Ser436 are not required for the flg22-induced RGS1 phosphorylation. Protoplasts expressing WT RGS1 or RGS1CT$^{S428A\ S435A\ S436A}$ were treated with flg22, and RGS1CT protein phosphorylation (band-shift) was detected by immunoblot analysis.

(c) BIK1 and PBL1 phosphorylate RGS1CT *in vitro*. RGS1CT-FLAG was expressed and purified from protoplasts, incubated with HIS-tagged BIK1, BAK1 or PBL1, and RGS1CT mobility was detected by immunoblot analyses.

The experiments in b and c were performed twice with similar results.
