# Supplementary material for: Ligand-triggered de-repression of Arabidopsis heterotrimeric G proteins coupled to immune receptor kinases
Source: Cell Res. 2018 Mar 15;28(5):529–43. doi: 10.1038/s41422-018-0027-5 (PMC5951851; doi:10.1038/s41422-018-0027-5)
Supplement: Supplementary file 7 — Supplementary figure S7(PDF 194 kb) [file 41422_2018_27_MOESM7_ESM.pdf]

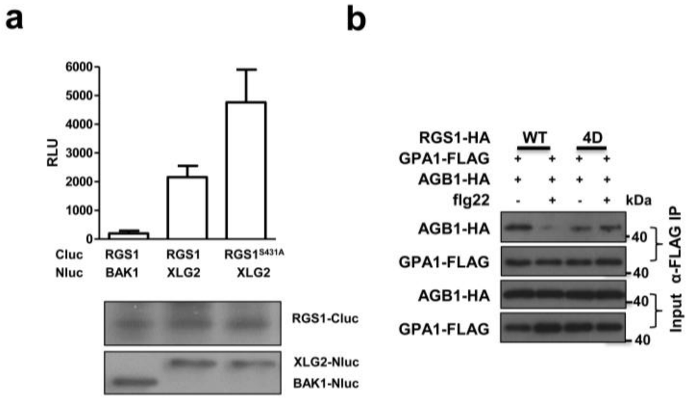

Supplementary information, Figure S7. RGS1 Ser431 phosphorylation site modulates RGS1-XLG2 interaction

(a) The RGS1^{S431A} mutant displays stronger interaction with XLG2. The indicated constructs were expressed in *N. benthamiana,* and luciferase complementation assays were performed. The strength of protein-protein interaction was expressed as arbitrary relative luminescence units (RLU, means $\pm$ SD; $n \geq 6$). Immunoblots indicate accumulation of proteins.

(b) Phospho-mimicking mutations in RGS1 Ser428/431/435/436 is sufficient to trigger GPA1-AGB1 dissociation. GPA1-FLAG and AGB1-HA were co-expressed in protoplasts along with RGS1-HA or RGS1^{4D}-HA (S428D, S431D, S435D and S436D). Protoplasts were then treated with or without flg22, and GPA1-AGB1 interaction was detected by co-IP assays.

The experiments were performed twice with similar results.
