# Supplementary material for: Ligand-triggered de-repression of Arabidopsis heterotrimeric G proteins coupled to immune receptor kinases
Source: Cell Res. 2018 Mar 15;28(5):529–43. doi: 10.1038/s41422-018-0027-5 (PMC5951851; doi:10.1038/s41422-018-0027-5)
Supplement: Supplementary file 8 — Supplementary figure S8(PDF 99 kb) [file 41422_2018_27_MOESM8_ESM.pdf]

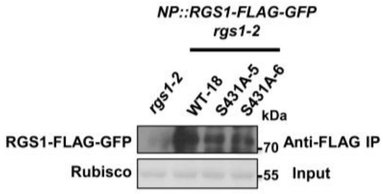

Supplementary information, Figure S8. RGS1 protein accumulation in trasngenic lines. The *RGS-FLAG-GFP* transgenic plants shown in Figure 7c and 7d were subjected anti-FLAG immunoprecipitation followed by anti-FLAG immunoblot analysis.
