# Supplementary material for: Ligand-triggered de-repression of Arabidopsis heterotrimeric G proteins coupled to immune receptor kinases
Source: Cell Res. 2018 Mar 15;28(5):529–43. doi: 10.1038/s41422-018-0027-5 (PMC5951851; doi:10.1038/s41422-018-0027-5)
Supplement: Supplementary file 9 — Supplementary table S1(PDF 102 kb) [file 41422_2018_27_MOESM9_ESM.pdf]

Table S1. Primers used.

Name	Primer
RGS1-kpnI-pucF	CACCGGTACCATGGCGAGTGGATGTGCTCT
RGS1-Csp45I-pucR	GGCTTCGAAACCGGGACTACTGCATCTGG
RGS1CT-kpnI-pucF	CACCGGTACCATGAACCAACCTCTTCTTTC
RGS1-SalI-pucR	GGCGTCGACACCGGGACTACTGCATCTGG
RGS1-S431A-F	AGGAAGGATACAGTTTTTCAGCTCCAAGACTGAGTTCAGTTCA
RGS1-S431A-R	TGAACTGAACTCAGTCTTGGAGCTGAAAAACTGTATCCTTCCT
RGS1-S428A-F	GCAATGCATAAGGAAGGATACGCTTTTTCATCTCCAAGACTGAGT
RGS1-S428A-R	ACTCAGTCTTGGAGATGAAAAAGCGTATCCTTCCTTATGCATTGC
RGS1-S435/436A-F	AGTTTTTCATCTCCAAGACTGGCTGCAGTTCAAGGCTCTGATGATCCT
RGS1-S435/436A-R	AGGATCATCAGAGCCTTGAACTGCAGCCAGTCTTGGAGATGAAAAACT
RGS1-450/452/453A-F	GATCCTTTCTATCAAGAACATATGGCAAAGGCTGCCAGATGCAGTAGTCCCGGTTTCG
RGS1-450/452/453A-R	CGAAACCGGGACTACTGCATCTGGCAGCCTTTGCCATATGTTCTTGATAGAAAGGATC
RGS1-430A-F	GCATAAGGAAGGATACAGTTTTGCAtCTCCAAGACTGAGTTCAGTTCA
RGS1-430A-R	TGAACTGAACTCAGTCTTGGAGaTGCAAAACTGTATCCTTCCTTATGC
RGS1CT-BamHI-GST-F	GCCGGATCCATGAACCAACCTCTTCTTT
RGS1CT-SalI-GST-R	CCGGTCGACTTAACCGGGACTACTGCAT
RGS1CT- HIS-F	AACCAACCTCTTCTTTCACAAATCAGCT
RGS1CT-SalI-HIS-R	CCGGTCGACACCGGGACTACTGCATCTG
LYK5KD-HIS-F	GGAGATATACCATGGCATGCTTCTACAAACGAAGGTCTA
LYK5KD-HIS-R	GTGGTGGTGCTCGAGGTTGCCAAGAGAGCCGGAACGA
EFR-Nluc-F	ACGGGGGACGAGCTCGGTACC ATGAAGCTGTCCTTTTCACTTG
EFR-Nluc-R	AACATCGTATGGGTAGTCGACCATAGTATGCATGTCCGTATTT
LYK5-Nluc-F	ACGGGGGACGAGCTCGGTACC ATGGCTGCGT GTACACTCCA CG
LYK5-Nluc-R	AACATCGTATGGGTAGTCGAC GTTGCCAAGA GAGCCGGAAC GA
CERK1-Nluc-F	ACGGGGGACGAGCTCGGTACC ATGAAGCTAAAGATTTCTCTAA
CERK1-Nluc-R	AACATCGTATGGGTAGTCGACCCGGCCGGACATAAGACTGACT
XLG2-(469-477)-F	TGCTTAACAAGCTTCTTTTGATTATTTACAAACAAGCAAGGTCTCT
XLG2-(469-477)-R	AGAGACCTTGCTTGTTTGTAAATAATCAAAAGAAGCTTGTTAAGCA
XLG2-(624-632)-F	CAGAATACGATCCTTCTGATATGTCTATGGAAGGACTTTCTTGTGT
XLG2-(624-632)-R	ACACAAGAAAGTCCTTCCATAGACATATCAGAAGGATCGTATTCTG
XLG2-(669-673)-F	CAGACATGAAGTACCAACTCATCAGCCTTGGTGAAAACTGGAAATT
XLG2-(669-673)-R	AATTTCCAGTTTTCACCAAGGCTGATGAGTTGGTACTTCATGTCTG
XLG2-(741-744)-F	ACAAAAGGTTTCTTTTGGTTCTACTCCTAGAAGAAAAGATCGAAGA
XLG2-(741-744)-R	TCTTCGATCTTTTCTTCTAGGAGTAGAACCAAAAGAAACCTTTTGT
RGS1-S431A-F	AGGAAGGATACAGTTTTTCAGCTCCAAGACTGAGTTCAGTTCA
RGS1-S431A-R	TGAACTGAACTCAGTCTTGGAGCTGAAAAACTGTATCCTTCCT
GPA1-kpn-pucF	GCCGGTACCATGGGCTTACTCTGCAGTA
GPA1-kpn-pucR	CGGTTCGAATAAAAGGCCAGCCTCCAGT
GPA1-(45-52)-F	TTCGGAAGCTTTTGCTACTTACAATTTTTAAGCAGATAAA
GPA1-(45-52)-R	TTTATCTGCTTAAAAATTGTAAGTAGCAAAAGCTTCCGAA
GPA1-(353-355)-F	CATCCTTCATGCTGTTCTTGATATTTGAGAAGAAAGTTCT

GPA1-(353-355)-R	AGAACTTTCTTCTCAAATATCAAGAACAGCATGAAGGATG
GPA1-NdeI-HIS-F	GCGCATATGGGCTTACTCTGCAGTAGAAG
GPA1-XhoI-HIS-R	GACCTCGAGTCATAAAAGGCCAGCCTCCA
AT3G18250-RTF	GAAGATGTAATGAGCATGGCCA
AT3G18250-RTR	GTGCCTGAAGTGGCTCTGTTG
BIP3-RTF	CACAGCGAAGATGACGAGGAA
BIP3-RTR	GATCGATCCCTATCACCGTCC
NHL6-RTF	ATCCGGTCCAAGATCCAGAAG
NHL6-RTR	CGTGTAGCAAAAGCATCTGCA
ACT8-F	ATGGCCGATGCTGATGACAT
ACT8-F	TTAGAAGCATTTTCTGTGGA
