# Supplementary material for: Ligand-triggered de-repression of Arabidopsis heterotrimeric G proteins coupled to immune receptor kinases
Source: Cell Res. 2018 Mar 15;28(5):529–43. doi: 10.1038/s41422-018-0027-5 (PMC5951851; doi:10.1038/s41422-018-0027-5)
Supplement: Supplementary file 10 — Supplementary table S2(PDF 116 kb) [file 41422_2018_27_MOESM10_ESM.pdf]

Table S2. RGSCT residues phosphorylated by BIK1 and PBL1 *in vitro*.

RGS1CT-HIS was co-expressed with GST-BIK1^{K105E}, GST-BIK1 or GST-PBL1 *in E. coli*, and the purified RGS1CT-HIS protein was subject to LC-MS/MS analysis.

Kinase	Phospho-peptide sequence	Position
BIK1^{K105E}	none	none
	MGQALGIPD$_p$SGLLFR	S278
	MGQALGIPD$_p$SGLLFRK	S278
	FKEEESCHEAMHKEGY$_p$SFSSPR	S428
	FKEEE$_p$SCHEAMHKEGYSFSSPR	S417
	FKEEESCHEAMHKEGYSF$_p$(SS)PR	S430/431
	FKEEESCHEAMHKEGY$_p$SF$_p$(SS)PR	S428, S430/431
BIK1	EGYSF$_p$(SS)PR	S430/431
	EGY$_p$SFSSPR	S428
	EGYSF$_p$(SS)PR	S430/431
	EGY$_p$SF$_p$(SS)PR	S428, S430/431
	LSSVQGSDDPFYQEHM$_p$SK	S450
	LSSVQGSDDPFYQEHMSKS$_p$SR	S453
	LSSVQGSDDPFYQEHMSK$_p$SSR	S452
	MGQALGIPD$_p$SGLLFR	S278
	MGQALGIPD$_p$SGLLFRK	S278
	FKEEESCHEAMHKEGY$_p$SFSSPR	S428
	FKEEESCHEAMHKEGYSF$_p$(SS)PR	S430/431
	FKEEESCHEAMHKEGY$_p$SF$_p$(SS)PR	S428, S430/431
	FKEEESCHEAMHKEGYSF$_p$(SS)PR	S430/431
	FKEEESCHEAMHKEGY$_p$SF$_p$(SS)PR	S428, S430/431
PBL1	EGYSF$_p$(SS)PR	S430/431
	EGY$_p$SFSSPR	S428
	EGYSF$_p$(SS)PR	S430/431
	EGY$_p$SF$_p$(SS)PR	S428, S430/431
	LSSVQGSDDPFYQEHM$_p$SK	S450
	LSSVQGSDDPFYQEHMSKS$_p$SR	S453
	LSSVQGSDDPFYQEHMSK$_p$SSR	S452

(Ser430 and Ser431 cannot be precisely differentiated by the LC-MS/MS data.)
